# A Fully Automated Pipeline for Normative Atrophy in Patients with Neurodegenerative Disease

**DOI:** 10.3389/fneur.2017.00727

**Published:** 2018-01-24

**Authors:** Christian Rummel, Fabian Aschwanden, Richard McKinley, Franca Wagner, Anke Salmen, Andrew Chan, Roland Wiest

**Affiliations:** ^1^Support Center for Advanced Neuroimaging (SCAN), University Institute for Diagnostic and Interventional Neuroradiology, Inselspital Bern, University of Bern, Bern, Switzerland; ^2^Department of Neurology, Inselspital Bern, University of Bern, Bern, Switzerland

**Keywords:** structural magnetic resonance imaging, automated morphometry, multiple sclerosis, atrophy progression, individualized medizine

## Abstract

**Introduction:**

Volumetric image analysis to detect progressive brain tissue loss in patients with multiple sclerosis (MS) has recently been suggested as a promising marker for “no evidence of disease activity.” Software packages for longitudinal whole-brain volume analysis in individual patients are already in clinical use; however, most of these methods have omitted region-based analysis. Here, we suggest a fully automatic analysis pipeline based on the free software packages FSL and FreeSurfer.

**Materials and methods:**

Fifty-five T1-weighted magnetic resonance imaging (MRI) datasets of five patients with confirmed relapsing–remitting MS and mild to moderate disability were longitudinally analyzed compared to a morphometric reference database of 323 healthy controls (HCs). After lesion filling, the volumes of brain segmentations and morphometric parameters of cortical parcellations were automatically screened for global and regional abnormalities. Error margins and artifact probabilities of regional morphometric parameters were estimated. Linear models were fitted to the series of follow-up MRIs and checked for consistency with cross-sectional aging in HCs.

**Results:**

As compared to leave-one-out cross-validation in a subset of the control dataset, anomaly detection rates were highly elevated in MRIs of two patients. We detected progressive volume changes that were stronger than expected compared to normal aging in 4/5 patients. In individual patients, we also identified stronger than expected regional decreases of subcortical gray matter, of cortical thickness, and areas of reducing gray–white contrast over time.

**Conclusion:**

Statistical comparison with a large normative database may provide complementary and rater independent quantitative information about regional morphological changes related to disease progression or drug-related disease modification in individual patients. Regional volume loss may also be detected in clinically stable patients.

## Introduction

Recently, the “no-evidence of disease activity” criteria (NEDA-3) have been extended (NEDA-4) in order to add cerebral atrophy as a potential surrogate biomarker for progression of multiple sclerosis (MS). Data from the FREEDOMS core and extension trials revealed that NEDA-4 status in the first year is a better predictor of long-term outcomes than NEDA-3. Volumetry has gained increasing attention as a possible approach to enable earlier and more accurate prognosis (1) and as a secondary outcome measure in clinical trials (2). Loss of brain tissue due to the progression of the disease may be easily overlooked during expert reading of magnetic resonance imaging (MRI). Monitoring of disease progression and therapy control may profit from quantitative volumetric approach to estimate lesion load and brain atrophy. Novel automatic methods for whole-brain volumetry (3–8) enable robust and effective longitudinal monitoring of atrophy and disease progression, while treatment goals in MS have shifted to require the setting of novel targets for disease monitoring.

Voxel-based morphometric (VBM) and surface-based morphometric group studies in MS patients (9–15) identified reduced mean overall cortical thickness and regional subcortical and cerebellar gray matter (GM) volume loss. Focal cortical thinning in frontal and temporal brain regions with a tendency to predominantly affect the left hemisphere was observed after first onset even in subgroups with mild disability. After relapses, in more severely disabled patients, cortical thinning was also evident in the pre- and postcentral gyrus. The presence of widespread cortical thinning has been suggested as a predictor for cognitive impairment, while atrophy of the superior frontal gyrus, the thalamus, and the cerebellum were independent predictors for conversion of clinically isolated syndrome (CIS) patients to MS (16). Reduced white matter (WM) volume and increased curvature of the cortical band has also been detected earlier in CIS (17). A follow-up study revealed significantly increased loss of total GM in MS patients with disability progression after 5 years (67 patients) as compared to patients with stable disability. Similar results were obtained for cortical GM and the volume of the putamen, whereas no difference was found in WM atrophy progression. At 10 years (50 patients) only a trend toward larger loss of total GM volume in patients with disability progression was detected (18).

Ahead of translating such findings into clinical practice, further research needs to address the extent and reproducibility of regional morphometric alterations in individual patients (i.e., whether they may contribute to subgroup classification and diagnostics). In this study, we suggest an automated analysis pipeline and statistical framework allowing the generation of morphometric reports that can be integrated into the diagnostic workup. Regional morphometric parameters estimated from single T1-weighted MRI datasets of individual MS patients are statistically compared to 323 healthy control (HC) datasets with a wide age range. The analysis is built on the free software packages FSL and FreeSurfer, yet the concept is not necessarily restricted to these environments. After testing the pipeline on subsets of the HC group, we provide a first proof-of-concept using 55 follow-up MRIs in five patients with a confirmed diagnosis of relapsing–remitting MS (RR-MS) and mild to moderate disability.

## Materials and Methods

The study was approved by the Kantonale Ethikkommission Bern. All subjects provided written informed consent and the study was performed in accordance with the Declaration of Helsinki.

### Patients

We applied our methodology to 55 consecutive MRI scans in five patients with RR-MS under treatment with natalizumab (Biogen Corp., Cambridge MA, USA). Inclusion criterion was that patients had at least ten follow-up MRI exams at our institution, covering at least 3 years of observation time. Overall, follow-up time was 5.03 ± 0.83 years (mean and SD) and the time interval between scans was 0.55 ± 0.06 years. Demographic patient information is provided in Table 1. Scanner specifications and sequences are compiled in Table S1 in Supplementary Material.

**Table 1:** Demographic and clinical information on patients.

	Pat. 1	Pat. 2	Pat. 3	Pat. 4	Pat. 5
**Sex**	Female	Female	Female	Female	Female
**Age (years)**					
At first scan	16.32	29.93	31.72	21.43	41.41
At last scan	22.19	35.24	36.78	25.09	46.67
Mean follow-up intervals (years)	0.65	0.48	0.56	0.52	0.53
Disease duration at first scan	4.33	n.a.	12.42	2.75	4.5
**New lesions**	+1 (TP2)	+1 (TP2)	+2 (TP7)	+1 (TP7)	+2 (TP2)
**EDSS**					
At first scan	1.5	n.a.	2	1.5	2
At last scan	1	n.a.	4	1	2
Annual change rate	−0.087	n.a.	0.435	−0.169	0.067
Rate different from zero	*p* = 0.388	n.a.	*p* = 0.395	*p* = 0.386	*p* = 0.567
Spearman correlation with age	*p* = 0.246	n.a.	***p* < 10^−4^**	***p* = 0.036**	*p* = 0.266
**MR scanner**	Verio	Verio	Verio	Verio	Verio
**MR sequence**					
MDEFT	0	1	1	2	0
MP-RAGE van der Kouwe	10	12	10	8	11

*EDSS, expanded disability status scale; MDEFT, modified-driven equilibrium Fourier transform; MP-RAGE, magnetization-prepared rapid gradient-echo; “+*n* (TPm)” means that *n* new lesions were discovered at time point *m* of the follow-up series, which were not present at time point *m*-1*.

*Values with significance p < 0.05 are displayed in bold-face*.

### Healthy Controls

**Figure 1:**
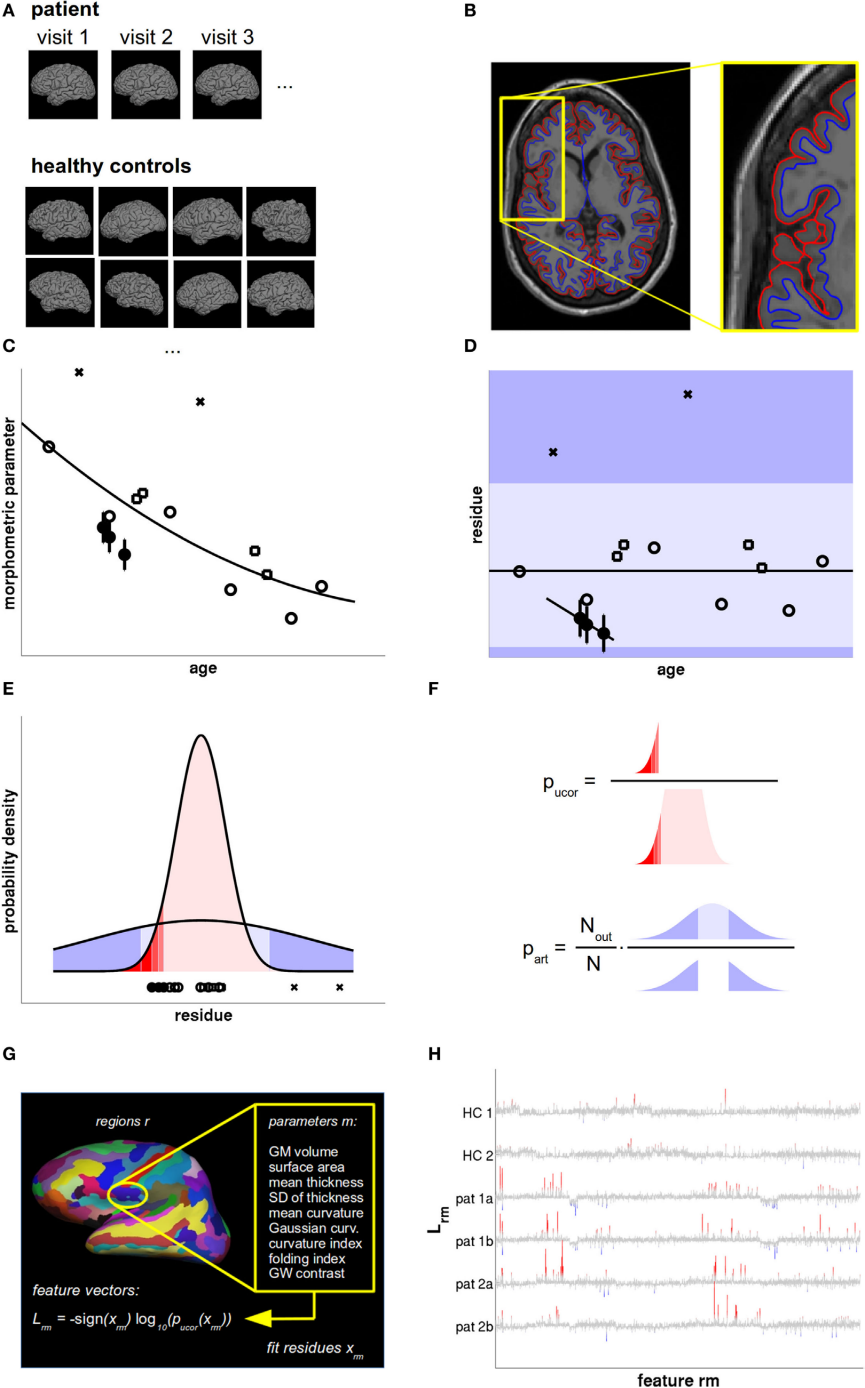
Data analysis and statistical evaluation, see Section “Materials and Methods” for details. **(A)** T1w magnetic resonance imaging (MRI) acquired during all patient visits were compared with a normative database of a large number of healthy controls (HCs), accounting for age, sex, and scanner parameters. **(B)** The MRIs were processed with FreeSurfer to define the pial (red) and gray–white surface (blue). From these, the morphometric parameters of interest were calculated region-wise. **(C)** The cross-sectional age dependence of morphometric parameters in HCs (open symbols) was fitted by a low-order polynomial. The overall measurement accuracy was estimated from HCs with follow-up MRIs within short temporal separation (open squares) and displayed as error bars in patient data (filled circles). **(D)** From the fit residues outliers were rejected (crosses) and linear age trends were fitted to the patient data. The dark shaded areas symbolize the regions where artifacts could be detected as outliers. Artifacts producing small values (light shaded region) remained undetected. **(E)** Valid measurements (red) and artifacts (blue) were both modeled as Gaussians with different widths. **(F)** The uncorrected *p*-value p_ucor_ of an observation in patients was calculated from the distribution of fit residues in HCs. The artifact probability p_art_ was estimated from a scaled empirical outlier fraction. The scaling factor was determined from the fraction of artifacts detectable as outliers. **(G)** Feature vectors were generated as the signed base-10 logarithm of 2,976 *p* values calculated per cortical region and morphometric parameter. **(H)** Feature vectors of two HCs and two follow-up magnetic resonance imagings (MRIs) of two multiple sclerosis (MS) patients as a function of feature number. Insignificant features (*p* > 0.01 uncorrected) are displayed in gray. Increased values are displayed in red and decreased values in blue, with full color indicating that deviations are significant after false discovery rate (FDR) correction.

Patient MRIs were statistically compared to a normative dataset (Figure 1A) consisting of HCs that were acquired during previous studies performed at the Inselspital, Bern, see Table S2 in Supplementary Material. After calculation of the region-specific morphometric parameters, only age, sex, an anonymized subject ID and MRI parameters (manufacturer, model, and sequence) were stored in the fully anonymized database as confounding variables. The data were derived from *N* = 323 high-resolution T1-weighted (T1w) imaging datasets from 267 neurologically healthy subjects (142 datasets from male subjects and 181 from females, mean age 35.9 years, SD 18.0 years, and age range 7–79 years, see Figure S1 in Supplementary Material for the age and sex distribution). For other demographic and technical parameters, see Table 2. Twenty-one participants were scanned twice, two were scanned three or four times, five were scanned five times, and one was scanned six times with separation smaller than 2 years.

**Table 2:** Demographic and technical parameters of the full control dataset, random, and patient-matched subsets used for leave-one-out cross-validation (LOOCV) and the multiple sclerosis (MS) patients.

	All healthy controls (HCs)	Randomly selected HCs	Patient-matched HCs	RR-MS patients
**Subject number**	267	31	18	5
**Dataset number**	323	34	19	55
**Age**				
Mean (SD)	35.9 (18.0)	35.6 (17.8)	27.8 (5.4)	30.9 (8.7)
Range	7–79	8–72	21–39	16–46
**Follow-up time (years)**				
Mean (SD)	n.a.	n.a.	n.a.	5.03 (0.82)
Interval, mean (SD)	n.a.	n.a.	n.a.	0.55 (0.06)
**Sex**				
Male	141	13	0	0
Female	182	21	19	55
**MR scanner**				
Verio	168	17	19	53
Trio	155	17	0	2
**MR sequence**				
MDEFT	171	18	3	4
MP-RAGE standard	62	7	0	0
MP-RAGE van der Kouwe	35	3	16	51
MP-RAGE ADNI	55	6	0	0

*The age and sex distribution of the control dataset is shown in Figure S1 in Supplementary Material*.

*ADNI, Alzheimer’s disease neuroimaging initiative; MDEFT, modified driven equilibrium Fourier transform; MP-RAGE, magnetization-prepared rapid gradient-echo; RR-MS, relapsing–remitting multiple sclerosis*.

We used subsets of our normative dataset to explore the expected rate of statistical anomalies in HCs by testing the selected subjects against the remainder in a leave-one-out cross-validation (LOOCV). One subset encompassed 34 randomly selected MRIs from 31 HCs (10% of all HCs, but at least one MRI from each previous study) and resembled the age, sex, MR type and sequence distribution of the whole normative dataset. To test whether deviations detected in patients depended on the different age, sex, scanner, and sequence characteristics in patients and controls (see Table 2), we also compiled a HC subset with characteristics matched to the MRIs of patients 2 and 3.

### Lesion Detection and Filling

Multiple sclerosis lesions may affect estimates of partial volume estimates (PVE) of GM, WM, and CSF (19). Similar biases are to be expected for volume segmentations. For cortical thickness measurements, the effect can be insignificant (20). Despite the recent finding that lesion filling might occasionally yield spuriously thinned cortex near juxta-cortical lesions (15), we integrated lesion filling ahead of morphometric analysis of MS patients. To detect lesions, we first co-registered fluid-attenuated inversion recovery (FLAIR) images acquired during the same session to the T1w images. We made use of Nabla Net (21), a recently published method using a deep convolutional architecture (a fully convolutional network in which an encoder–decoder structure computes high-level features that are combined with lower-level features using skip connections) and winning algorithm at the MICCAI 2016 MSSEG challenge to automatically produce lesion masks from FLAIR images. We calculated lesion volumes at each visit and used the lesion masks as inputs to FSL’s lesion_filling command to obtain T1w images with lesions filled by white-matter intensities. In HCs, these steps were not performed.

### Automated Processing of T1-Weighted Images

Processing of lesion filled (if applicable) T1w images was performed using the free software packages FSL and FreeSurfer on a quad-core workstation under Ubuntu Linux, release 14.04 LTS. For displaying statistics and results, self-written Octave scripts were used (CR). In the paper, two significance levels α = 0.01 and α = 0.05 are used and results are contrasted.

Details of the volumetric and morphometric analysis pipeline are provided in the Presentation S1 in Supplementary Material. In brief, the total volumes of cerebrospinal fluid (CSF), GM, and WM were estimated using FSL^1^ [version 5.0 (22)]. For estimation of the volumes of segmentations of the GM, WM, and CSF volume we used FreeSurfer^2^ (version 5.3.0). Regions of interest (ROIs) included segmentations of the hippocampus and the amygdala, the thalamus, the basal ganglia, the ventricles, the corpus callosum, and the cerebellum. The procedures are described in detail in Ref. (23, 24).

For surface-based analysis (SBA), the interfaces between WM and GM as well as between GM and CSF were estimated with FreeSurfer (Figure 1B). The technical details of these procedures have been described previously (25–27). Cortical ROIs were defined by automatic parcellation of the cortex (28) according to the atlases by Desikan et al. (29) and Destrieux et al. (30) and surface-based morphometric parameters were reported as parcellation-wise averages. Details on the extraction of the following nine regional volumetric and morphometric parameters are summarized in Presentation S1 in Supplementary Material: cortical thickness (mean and SD), cortical surface area, cortical GM volume, mean and Gaussian curvature of the cortex, curvature and folding index, and the contrast between GM and WM along the cortical band.

### Quality Control

FreeSurfer’s automatic surface tessellation, volume segmentation, and cortex parcellation procedures may occasionally produce errors, which in turn may decrease the accuracy of anomaly detection. This can be prevented either by labor-intensive visual quality control in each individual or by statistical rejection of outliers. To automatically reject outliers, values of raw morphometric parameters X in each ROI exceeding within-group thresholds X_<_ and X_>_ more than 1.5 interquartile ranges from the 25% and 75% percentiles of the full distribution were rejected automatically. After a provisional polynomial fit to the empirical age dependence (see Figure 1C and text below), the same procedure was repeated for the residues *x* = *X* − *X*_poly_. Then, a final age fit was performed on the cleaned data. In our large control group (*N* = 323), we limited artifact rejection to this automated processing (Figure 1D). In the smaller group of follow-up MRIs from the same patient (10 ≤ *N* ≤ 13, see Table 1), this rejection scheme was followed up by visual inspection of doubtful brain regions using FreeSurfer’s freeview software with the pial and gray–white surfaces overlayed over the T1w MRI. In rare occasions, measurements caused by artifacts were subsequently excluded manually.

After exclusion of statistical outliers from the HC dataset, the expected region- and parameter-specific measurement accuracies were estimated as the square root of the mean intra-subject squared error of the 87 MRI datasets from the *N*_rep_ = 31 healthy subjects who had undergone more than one MRI acquisition with separation smaller than 2 years, see Presentation S1 in Supplementary Material for details.

### Standardized Result Presentation

**Figure 2:**
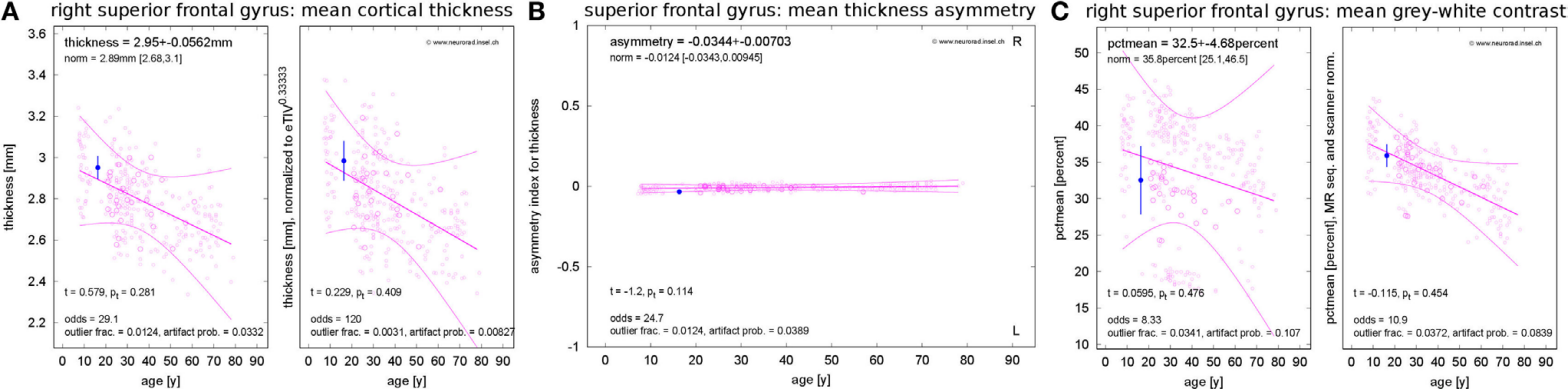
Standardized result display of morphometric parameters as a function of age using the example of the mean cortical thickness **(A)** and corresponding asymmetry index **(B)** as well as the mean percentage of gray–white contrast **(C)** in the right superior frontal gyrus (first visit of patient 1). The patient’s parameter estimates are shown as a filled symbol with error bars representing the parameter and region-specific estimated measurement reliability. The open symbols represent the values for the healthy controls (HCs). Large open symbols are controls matching the patient exactly for sex, MR scanner, and specific sequence; small open symbols have at least one mismatch. The best-fitting polynomial age trend (see text) and its confidence bounds are shown as solid lines. The left part of **(A,C)** reports the raw parameter values in physical units, whereas eTIV **(A)** or scanner-sequence normalized values **(C)** are shown in the right part. At the top of each panel, the parameter estimates are reported together with their estimated errors and the age-adjusted expectations in HCs (means and range). At the bottom, the test statistics and their significance are given. Also the odds for a valid versus erroneous measurements, the empirical outlier fractions and the estimated artifact probabilities are reported.

To support expert inspection of T1w MRIs and provide additional quantitative information, we displayed the patients’ morphometric parameters and their measurement accuracies in each ROI together with the normative values as a function of age, see Figure 2. We used an in-house written Octave script (CR) to generate a standardized result display for all morphometric parameters, all volume segmentations and all cortical parcellations without preselection. All results were stored as png figures and automatically generated html pages were used to allow unrestricted navigation and switching between region-based and measure-based result compilations.

Besides the raw morphometric parameters in physical units as estimated by FreeSurfer and FSL (left part of Figure 2A), brain volume normalized versions were evaluated (right part of Figure 2A). To this end, all parameters were scaled to the mean estimated total intracranial volume (eTIV) of the full control dataset as reported by FreeSurfer. The geometrically expected scaling exponents (31) were accounted for, i.e., volumes were scaled as eTIV^n^ with exponent *n* = 1, areas with exponent *n* = 2/3 and thicknesses as well as radii with exponent *n* = 1/3. According to their definition as functions of inverse radii, the mean curvature and the intrinsic curvature index were scaled with exponent *n* = −1/3 and the Gaussian curvature and the folding index were scaled with *n* = −2/3.

To assess inter-hemispheric asymmetries of morphometric parameters, we estimated asymmetry indices by calculating the difference between corresponding right and left structures and normalizing to their sum (Figure 2B). For complete symmetry, this index is zero and ranges between −1 for extremely left-dominated and +1 for extremely right-dominated structures.

The gray–white contrast is highly dependent on the acquisition sequence and the MR scanner type. For example, the MDEFT sequence produces higher contrast than MP-RAGE sequences. This can be seen in the left part of Figure 2C, where distinct point clouds of HC data are apparent, each one stemming from a different scanner–sequence combination. To compensate this influence, we normalized contrasts to the same mean value for each scanner–sequence combination rather than to mean eTIV (right part of Figure 2C).

Since morphometric parameters, and thus the significances of observations, are age-dependent (32–38), all results were displayed as a function of age. Polynomial age trends were fitted to the estimates of all *N* = 323 members of the normative dataset and statistical analysis was performed on the fit residues (Figures 1C–E). To avoid overfitting, the polynomial degree *d* was increased stepwise between zero (constant fit) and a maximal value of *d*_max_ until the higher degree ceased to reduce the variance of the residues significantly (*F*-test for nested models). We heuristically chose *d*_max_ = *N*/20 = 16 as an upper limit. A Bonferroni correction of the significance level α by a factor 1 ≤ *d* ≤ *d*_max_ was applied to account for the number of multiple tests actually required on step *d* of this procedure in the simplest possible way. The polynomial age trends and corresponding confidence intervals for the full cloud of the normative dataset were displayed together with the patients’ morphometric parameters; see Figure 2.

### Empirical Outlier Rate, Artifact Probabilities, and Odds for Valid vs. Erroneous Measurements

Despite outlier and artifact rejection (see above), the measurement of morphometric parameters may result in spurious values caused by image artifacts or too-weak gray–white contrast. We modeled residues *x* caused by artifacts by Gaussian probability densities centered at fit residue *x* = 0. The width σ_out_ was estimated by the mean absolute fit residues of all those HCs that were discarded as outliers (Figure 1E). Under these circumstances, the probability of finding artifact-related measurements as extreme as or even more extreme than the empirically detected outlier thresholds *x*_<_ and *x*_>_, i.e., the probability 0 ≤ *p*_out_ ≤ 1 of detecting artifacts as outliers, is given in terms of the cumulative density function of the normal distribution with width σ_out_:

(1) $$p_{\text{out}}=\Phi_{\sigma_{\text{out}}}\left(x_{<}\right)+1-\Phi_{\sigma_{\text{out}}}\left(x_{>}\right).$$

As artifacts may occur at any magnitude, some may result in parameter estimates within the range [*x*_<_,*x*_>_] and these cases remain undetected by our quality control procedures. To estimate the region- and parameter-specific artifact probability, we counted the empirical outlier numbers *N*_out_ found in the *N* = 323 HCs (see above) and scaled the outlier rate up by a factor 1/*p*_out_ (see Figure 1F):

(2) $$p_{\text{art}}=N_{\text{out}}/N*1/p_{\text{out}}.$$

We set *p*_art_ = 0 if for a morphometric parameter or ROI no outliers were observed in HCs, taking into account that this value is only a *lower* limit of the true artifact probability. From the artifact probability, we calculated the region- and parameter-specific odds for a valid as opposed to an erroneous (i.e., artifact-corrupted) observation by

(3) $$\text{odds}=(1-p_{\text{art}})\text{/}p_{\text{art}}$$

and trusted the observation when odds » 1.

### Statistical Assessment

Sex (38, 39), MR type (38, 40), and sequence (41) can bias morphometric estimates. To reduce confounding effects, the statistical significance of deviations between the patient and the controls was assessed by restricting the analysis to that subset of HCs who fully matched the patient’s characteristics with respect to sex, MR type, and acquisition sequence. The significance of an observed deviation of a patient’s fit residue *x* from the normative data can be influenced by three factors, (i) the probability of the observation being valid (as opposed to erroneous, i.e., caused by any kind of artifact), (ii) the width of the distribution of the normative data, and (iii) the measurement error of the observation itself. Treatment of issue (i) has already been discussed in the last paragraph. The larger the odds for valid measurement, the more trustworthy an observation. Issues (ii) and (iii) were treated by calculating the probability of finding an empirical observation at *x* if its true value *x*’ is uncertain with a measurement error σ_meas_. Assuming normally distributed fit residues in the subset of HCs used for statistics (mean at *x* = *x*_norm_ and SD σ_norm_), straightforward algebra yields that the variances add up σ^2^ = σ_norm_^2^ + σ_meas_^2^ and, thus, the *p* values are given by the cumulative density function Φ_σ_ of a Gaussian distribution with mean *x*_norm_ and SD σ in the following way (Figure 1F):

(4a) $$p_{\text{ucor}}(x)=1-\Phi_{\sigma }(x-x_{\text{norm}})\text{ if }x>x_{\text{norm}},$$

(4b) $$p_{\text{ucor}}(x)=\Phi_{\sigma }(x-x_{\text{norm}})\text{ if }x<x_{\text{norm}}.$$

### Feature Vectors

To assess the reproducibility of direction and magnitude of deviations *x*_rm_ in ROIs *r* and morphometric parameters *m*, we defined feature vectors by signed base-10 logarithms of *p* values (Figure 1G)

(5) $$L_{\text{rm}}\text{=}-\text{sign}(x_{\text{rm}})\cdot {\text{log}}_{\text{10}}(p(x_{\text{rm}})).$$

This yielded large values where the deviations were significant and small ones otherwise (Figure 1H). The overall length of the feature vectors was 2,976, see Table S3 in Supplementary Material.

### Estimation of Regional Atrophy Progression from Follow-up MRIs

The evaluation concept presented above can be extended to group studies (independent measurements) and follow-up analysis of individual patients (dependent measurements). If the results of more than two exams are available, one can test not only for different mean position of the fit residues *x* in patients and HCs but also for different age dependence. The first question can be answered by a two-sample *t*-test (variant for unequal group sizes and unequal variances). To address the age dependence, we fitted a linear model *x(t)* = *a⋆t* + *x_0_* to the patients’ residues *x* at time points *t*. If this model described the patients’ age trend well (i.e., the residual variance *S*^2^ was not larger than the measurement error σ_meas_^2^ in a chi-squared test on the variance), we performed a *z*-test to evaluate whether the slope *a* differed from zero. For *n* measurements, the slope uncertainty is given in terms of *S*^2^ and the variance σ_t_^2^ of the measurement time points by

(6) $$\Delta \text{a}=\frac{S}{\sigma_{t}}\cdot \frac{1}{\sqrt{n-2}}.$$

We used the same approach to assess the stability of the lesion volume or of the disability level (EDSS) during the follow-up period.

### Significance Highlighting

When generating the standardized result representation, deviations of the single patient estimate or of the longitudinal change during the follow-up period from the expectation were automatically highlighted with a yellow figure background if the *p* value was smaller than the significance level α. The highlighting indicated statistical anomalies that required secondary inspection by a trained expert. Correction for multiple comparisons during extensive testing (>10^3^ tests in each dataset) was performed using the concept of false discovery rate (FDR) (42). Deviations that remained significant after FDR correction were highlighted with a red figure background.

## Results

### Technical Parameters

Several morphometric parameters evaluated in cortical parcellations (cortical GM volume, cortical surface area, mean and SD of the cortical thickness, four curvature measures, and the gray–white contrast) turned out to be dependent on each other in the HC data. Figure S2 in Supplementary Material reveals a strong correlation of the cortical GM volume with mean thickness and the surface area, but these measures were only weakly correlated with one another. Similarly, the SD of the cortical thickness was correlated with the mean thickness and the GM volume. The four curvature parameters were highly correlated with each other and the gray–white contrast was weakly negatively correlated with them.

**Figure 3:**
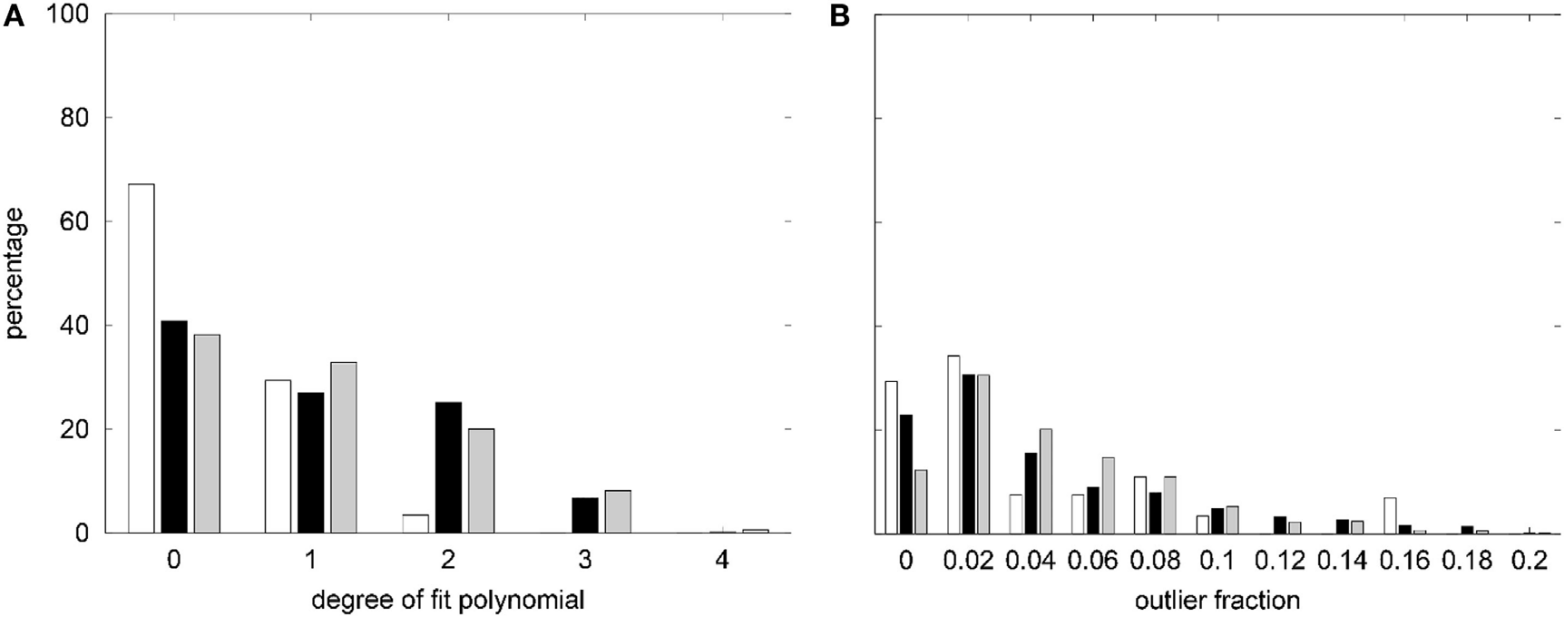
**(A)** Summary of the degree distribution of the best polynomial age fits to all morphometric parameters measured in the healthy controls (HCs). **(B)** Distribution of outlier fractions of morphometric parameters in HCs. White, asymmetry indices; black, raw parameters on both hemispheres; gray, parameters after normalization to estimated total intracranial volume (eTIV*^n^*) or scanner and sequence. Parameter-specific results are compiled in Figure S3 in Supplementary Material.

The degree distribution of best low-order polynomial fits to the age trends of asymmetries, raw, and eTIV or scanner–sequence normalized morphometric parameters in the normative dataset are displayed in Figure 3A. Despite allowing a maximal degree *d*_max_ = 16, constant (*d* = 0) and linear (*d* = 1) fits were sufficient in over 90% of asymmetry indices. For morphometric parameters on the left and right hemisphere, the same was true when quadratic fits were included. The maximal polynomial degree *d* = 4 was used in very rare cases and *d* = 3 was necessary in a higher fraction of cortical parcellations for the mean and the SD of cortical thickness than for GM volumes, cortical surface areas, and curvature measures (see Figure S3A in Supplementary Material).

**Figure 4:**
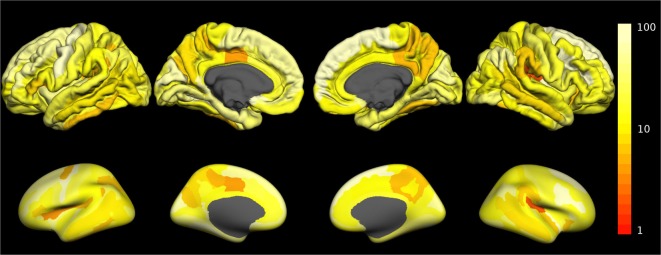
Spatial distribution of the odds for valid versus erroneous measurement of the mean cortical thickness. In the top row, the odds are displayed on the surface of a standard brain. To allow better visibility of regions located inside sulci, the same data are displayed on an inflated standard brain in the bottom row. The odds for other morphometric parameters are displayed in Figures S4–S11 in Supplementary Material.

The fraction of outliers found in the normative dataset is displayed in Figure 3B and a detailed compilation is given in Figure S3B in Supplementary Material. The largest mean outlier fractions were observed for curvature measures. The spatial distribution of odds for valid versus erroneous measurements is shown in Figure 4 for the example of the mean cortical thickness. Values larger than 10 were very common and the highest odds (i.e., highest confidence in valid measurements) were located on the frontal lobes. The lowest odds (i.e., largest likelihood for artifacts in cortical thickness) were found in the cingulate gyrus and the insula. Figures S4–S11 in Supplementary Material show the corresponding information for all other cortical morphometric parameters.

### Reproducibility of Feature Vectors

**Figure 5:**
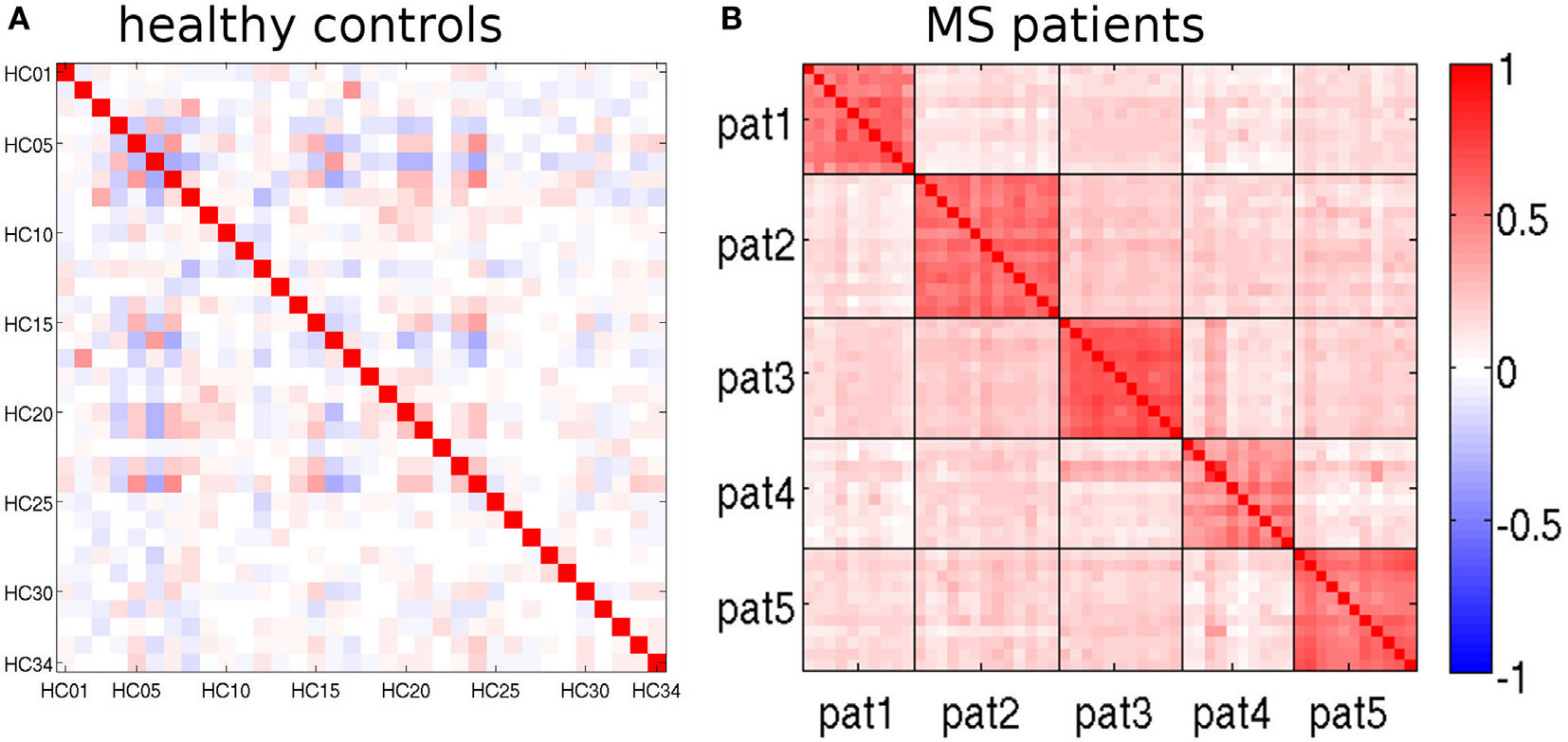
Pearson’s correlation matrices of the feature vectors L_rm_ (see text). **(A)** randomly selected leave-one-out cross-validation (LOOCV) test set and **(B)** multiple sclerosis (MS) patients. Magnetic resonance imaging (MRIs) belonging to the same patient are separated by black lines.

The feature vectors L_rm_ encode deviations from the age- and sex-adjusted expectation in HCs. Figure 5 shows Pearson’s correlation matrix between the L_rm_ calculated from different MRIs. In the HCs of the randomly selected LOOCV test set (Figure 5A), the correlation coefficients were predominantly small. Matrix elements connecting different MRI datasets of the same healthy subjects were among the largest ones (datasets 2 and 17, 3 and 8, as well as 6 and 16). By contrast, in the patient dataset (Figure 5B), a prominent block pattern became apparent, clearly reflecting patient-individual properties of the L_rm_. The observation that correlation between feature vectors of different patients was in general higher than between different HCs might be associated with disease-related effects in RR-MS that need further investigation in a larger cohort.

### Anomaly Detection in HCs

The overall rates of statistical anomalies with *p* < α in the random and patient-matched LOOCV subsets and the MRIs of patients 2 and 3 are reported in Table 3 for analysis at significance levels α = 0.01 and α = 0.05. We used binomial tests to check the null hypotheses that the anomaly rates were given by α, on the one hand, and by the empirical rate obtained in the random LOOCV subset on the other. At significance level α = 0.01, anomaly rates without FDR correction (yellow background in our standard result representation) were between 1.50 and 1.74% in HCs. This turned out significantly larger than expected (Bonferroni corrected for multiple comparisons). With the exception of the asymmetry indices this was not the case when repeating the analysis at a significance level α = 0.05. The overall anomaly rates in the patient-matched HCs (uncorrected for multiple comparisons) were not different from the ones empirically found in the randomly selected HCs. However, FDR-corrected anomaly detections (red background in our standard result representation) were smaller in the patient-matched HCs than in the random HC subset.

**Table 3:** Overall assessment of anomalies found in the randomly selected and the patient-matched LOOCV test sets as well as in the patient datasets at two significance levels α = 0.01 and α = 0.05.

			*p* < 0.01		*p* < 0.05	
		Test count	p_uncorr_	p_FDR_	p_uncorr_	p_FDR_
**LOOCV: 34 random healthy controls (HCs)**						
Raw	Count	67,558	1,088	343	3331	464
	Percentage		1.61%	0.51%	4.93%	0.69%
	p_bino (nominal)		**0**	n.a.	0.207	n.a.
	p_bino (empirical)		–	–	–	–

Estimated total intracranial volume (eTIV) and scanner/sequence normalized	Count	67,558	1,116	349	3548	462
	Percentage		1.65%	0.52%	5.25%	0.68%
	p_bino (nominal)		**0**	n.a.	0.001	n.a.
	p_bino (empirical)		–	–	–	–

Asymmetry	Count	33,524	582	56	2,244	114
	Percentage		1.74%	0.17%	6.69%	0.34%
	p_bino (nominal)		**0**	n.a.	**0**	n.a.
	p_bino (empirical)		–	–	–	–

**LOOCV: 19 matched HCs**						
Raw	Count	37,753	567	126	1985	164
	Percentage		1.50%	0.33%	5.26%	0.43%
	p_bino (nominal)		**0**	n.a.	0.012	n.a.
	p_bino (empirical)		0.048	**<*10^−6^***	0,002	**<*10^−9^***

eTIV and scanner/sequence normalized	Count	37,753	578	129	2,030	170
	Percentage		1.53%	0.34%	5.38%	0.45%
	p_bino (nominal)		**0**	n.a.	<10^−3^	n.a.
	p_bino (empirical)		0.033	**<*10^−6^***	0,140	**<*10^−8^***

Asymmetry	Count	18,734	279	17	1,274	25
	Percentage		1.49%	0.09%	6.80%	0.13%
	p_bino (nominal)		**<10^−9^**	n.a.	**0**	n.a.
	p_bino (empirical)		0.005	0.004	0.283	**<*10^−7^***

**Multiple sclerosis (MS) patients 2 and 3: 19 magnetic resonance imagings (MRIs)**						
Raw	Count	37,753	1460	528	3,533	736
	Percentage		3.87%	1.40%	9.36%	1.95%
	p_bino (nominal)		**0**	n.a.	**0**	n.a.
	p_bino (empirical)		**0**	**0**	**0**	**0**

eTIV and scanner/sequence normalized	Count	37,753	1,530	545	3,736	764
	percentage		4.05%	1.44%	9.90%	2.02%
	p_bino (nominal)		**0**	n.a.	**0**	n.a.
	p_bino (empirical)		**0**	**0**	**0**	**0**

Asymmetry	Count	18,734	879	183	2,256	329
	Percentage		4.69%	0.98%	12.04%	1.76%
	p_bino (nominal)		**0**	n.a.	**0**	n.a.
	p_bino (empirical)		**0**	**0**	**0**	**0**

*The randomly selected subset (34 MRI datasets) was tested against the expected error rate α (binomial tests). By contrast, the MS patient and patient-matched subsets were also tested against the empirical anomaly rates obtained in the randomly selected subset. *p* Values in bold-face indicate significantly enlarged and in italic significantly decreased values (after FDR correction for multiple comparisons). Specific results of morphometric parameters are compiled in Table S4 in Supplementary Material*.

*FDR, false discovery rate; LOOCV, leave-one-out cross-validation; n.a., not applicable*.

When comparing patient MRIs to LOOCV in HCs, the anomaly rates were elevated by at least a factor of 2. *All* differences were highly significant with *p* = 0 to machine precision. Anomaly rates for all morphometric parameters separately are compiled in Table S4 in Supplementary Material. With the exception of the PVE (and to a lesser extent also of the SDs of the cortical thickness) anomaly rates were elevated in the patients for all morphometric parameters. This effect was less prominent when anomaly detection was FDR-corrected to account for multiple comparisons. In healthy subjects, the rate of (potentially false) detections was higher for the Gaussian curvature, the curvature and the folding index than for the other morphometric parameters.

### Exemplary Use Case: Progressive Volume Loss in Patients with Relapsing–Remitting Multiple Sclerosis

A study evaluating the usefulness of the proposed analysis pipeline for single time-point MRIs in a larger number of temporal lobe epilepsy patients is presented elsewhere (43). Here, we concentrate on progressive volumetric and morphometric changes detectable in individual patients with RR-MS. During observation time all patients had only a small amount of new lesions (Table 1) and none of the slopes of linear fits to the lesion volumes estimated from the FLAIR images was statistically different from zero (Table 4). The slope of a linear fit to the EDSS disability index was not different from zero, either (Table 1). However, the Spearman correlation coefficient of EDSS with age was significant for patients 3 and 4.

**Table 4:** Partial volume estimates (PVE) at first and last magnetic resonance imaging (MRI) exam and annual rate of volume change.

	Pat. 1		Pat. 2		Pat. 3		Pat. 4		Pat. 5	
	(ml)	*p*-Value	(ml)	*p*-Value	(ml)	*p*-Value	(ml)	*p*-Value	(ml)	*p*-Value
**GM volume (ml)**										
First MRI	630	0.08	514	−0.494	514	0.462	**565**	**−0.008**	588	0.073
Last MRI	538	−0.284	503	0.485	511	0.463	527	−0.088	574	0.077
Annual change rate	**−11.5**	**−0.003**	−5.2	−0.142	**−5.4**	**−0.036**	**−11.8**	**−0.032**	−2.9	−0.209

**WM volume (ml)**										
First MRI	492	−0.207	446	−0.256	463	−0.484	496	0.213	499	−0.231
Last MRI	480	0.483	438	−0.206	464	−0.448	473	−0.294	496	−0.262
Annual change rate	−2.8	0.136	−1	0.434	**2.1**	**0.046**	3.4	0.057	0	0.296

**CSF volume (ml)**										
First MRI	236	−0.286	259	0.19	245	−0.49	289	0.174	283	0.481
Last MRI	245	0.429	269	0.101	257	0.377	**285**	**0.034**	284	0.468
Annual change rate	**2.8**	**4 × 10^−4^**	**2.5**	**0.043**	**0.9**	**0.025**	−1.4	−0.302	**1.4**	**0.047**

**Brain volume (ml)**										
First MRI	1,120	0.286	959	−0.19	977	0.49	1060	−0.174	1,090	−0.481
Last MRI	1,020	−0.429	941	−0.101	975	−0.377	**1,000**	**−0.034**	1,070	−0.468
Annual change rate	**−14.2**	**−4 × 10^−4^**	**−6.3**	**−0.043**	**−3.3**	**−0.025**	−8.4	0.302	**−2.9**	**−0.047**

**Lesion volume (ml)**										
First MRI	7.08	n.a.	11.3	n.a.	13.12	n.a.	8.39	n.a.	4.04	n.a.
Last MRI	5.74	n.a.	11.31	n.a.	14.28	n.a.	8.42	n.a.	3.19	n.a.
Annual change rate	−0.05	−0.388	−0.03	−0.401	0.12	0.536	0.5	0.774	−0.07	−0.389

*Values with significance p < 0.05 after eTIV correction are displayed in bold-face. The sign of the p-value indicates whether the observed deviation was larger or smaller than expected*.

*CSF, cerebral-spinal fluid; GM, gray matter; WM, white matter*.

**Figure 6:**
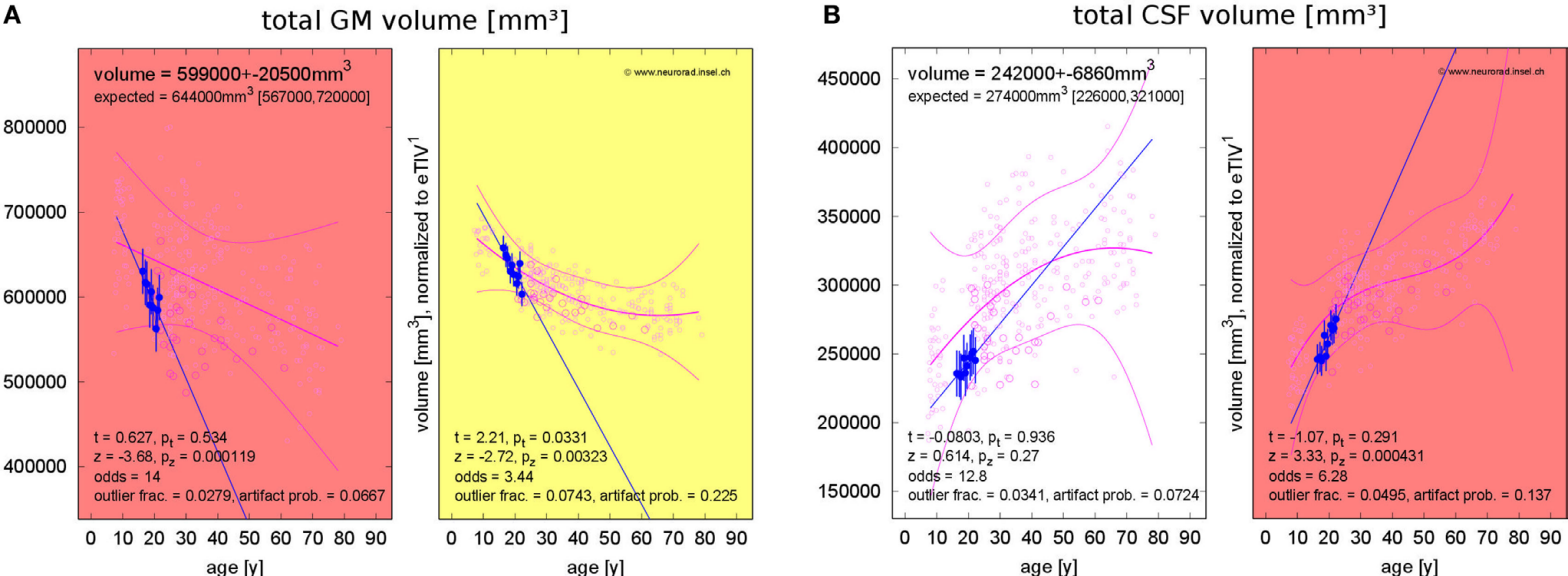
Progressing atrophy in patient 1 over 5.9 years [10 magnetic resonance imaging (MRI) exams]. **(A)** Exceeding the expected decrease of gray matter (GM) volume. **(B)** Exceeding the expected increase of cerebrospinal fluid (CSF) volume after correction for estimated total intracranial volume (eTIV).

Table 4 summarizes results for the PVE of all patients. With the exception of patient 4, none of the PVEs deviated significantly from the age-adjusted expectation (significance level α = 0.05), neither at the first nor at the last MRI exam of the observation period. In patient 4, we detected reduced GM volume for a female of 21 years at the first MRI exam (*p* = 0.008). At the last exam, brain volume was too small and CSF volume too large (both *p* = 0.034) for a female of 25 years. PVE *changes* were often significantly larger than expected from cross-sectional aging in the control group. Brain volume loss and CSF volume increase were stronger than expected in 4/5 patients and GM loss was stronger than expected in 3/5 patients. Examples of GM volume loss and CSF volume increase in patient 1 are presented in Figure 6. Indeed, the volumes were still within the normal ranges, but progressive decline over the past 6 years deviated from the expectation (*p* = 0.003 for GM and *p* < 10^−3^ for CSF, both after normalization for eTIV).

**Figure 7:**
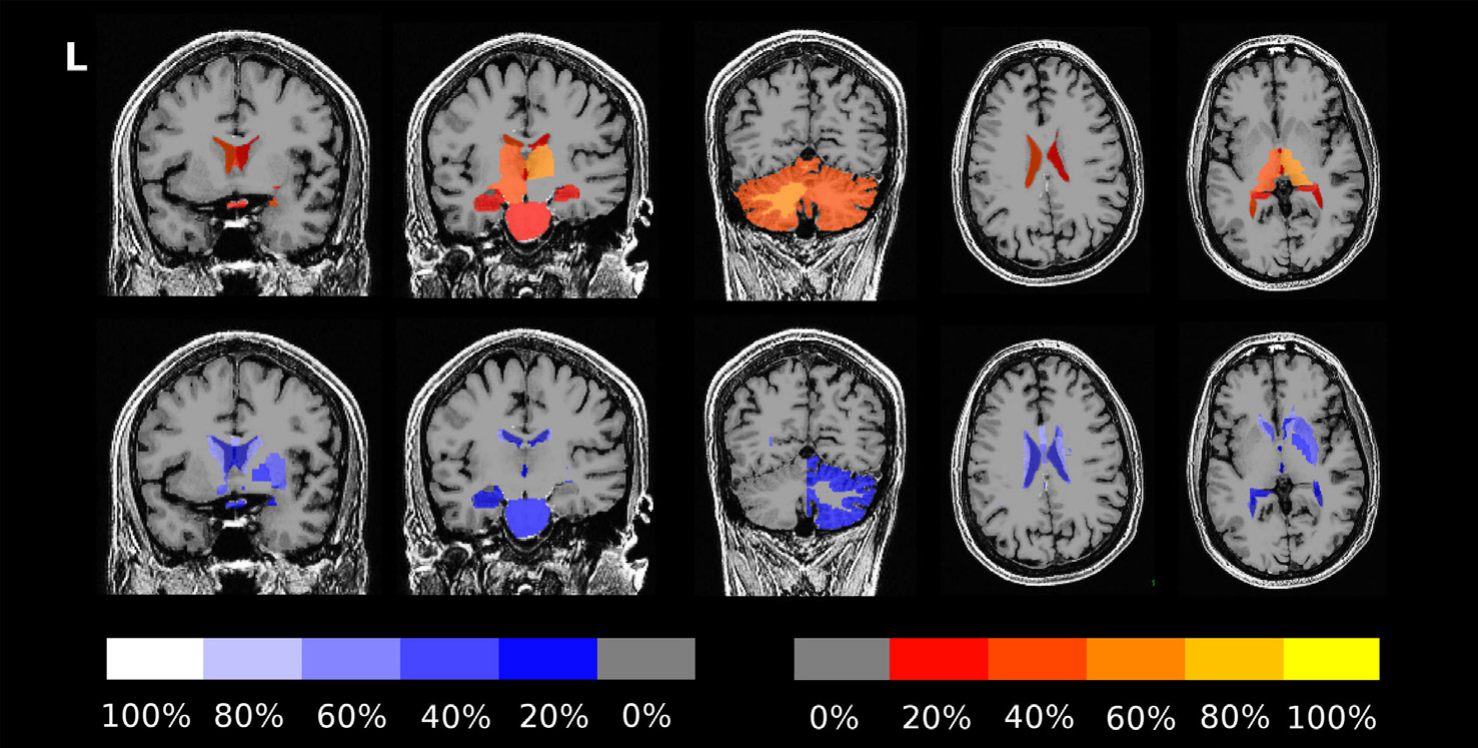
Percentage of patients showing more severe than expected linear changes of volume segmentations during the follow-up period. The top row shows deviations toward stronger increase or weaker decrease on a red-to-yellow color scale. The bottom row shows deviations toward stronger decrease or weaker increase on a blue-to-white color scale. The images are in neurological orientation, i.e., the left side of the images correspond to the left hemisphere.

**Figure 8:**
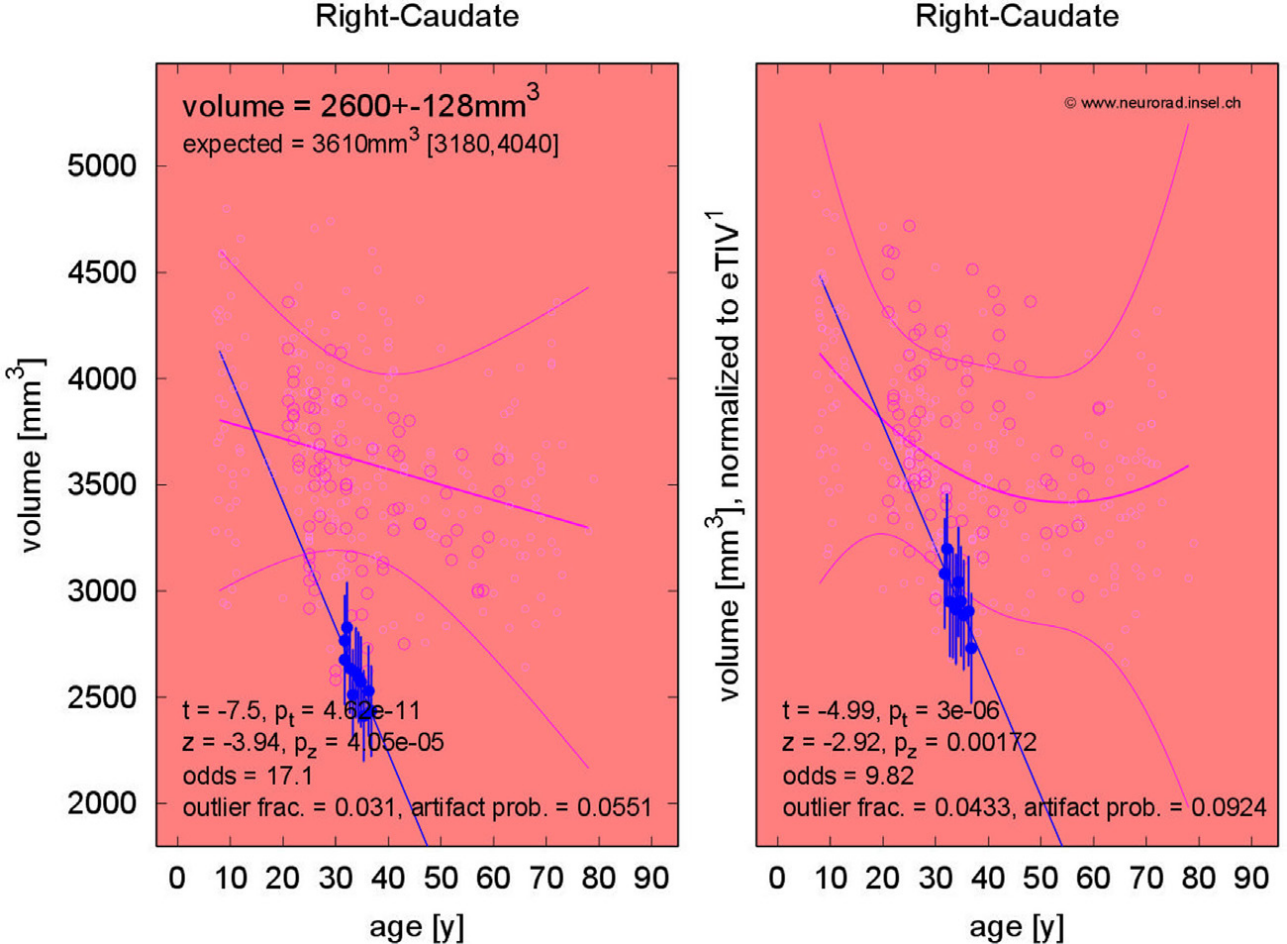
Progressive volume loss of the right caudate in patient 3.

The fraction of patients presenting with deviations of volume segmentations, of mean cortical thickness, or of the gray–white contrast deviations of from age- and sex-adjusted expectations in the first and last MRI is compiled in Figures S12–S17 in Supplementary Material. Here, we focus on more severe than expected linear *changes* of these parameters. For the volumes of the lateral ventricles, the brain stem, and the right-hemispheric cerebellar GM, we observed deviations from the expected change rate in both directions, see Figure 7. Note that the blue-to-white color scale can either indicate significantly stronger than expected decrease or weaker than expected increase and vice versa for the red-to-yellow colorbar. An example of more severe than expected volume loss of the right caudate volume (found in 4/5 patients, same for the left hemisphere) in patient 3 is given in Figure 8 (*p* = 0.002 after normalization for eTIV).

**Figure 9:**
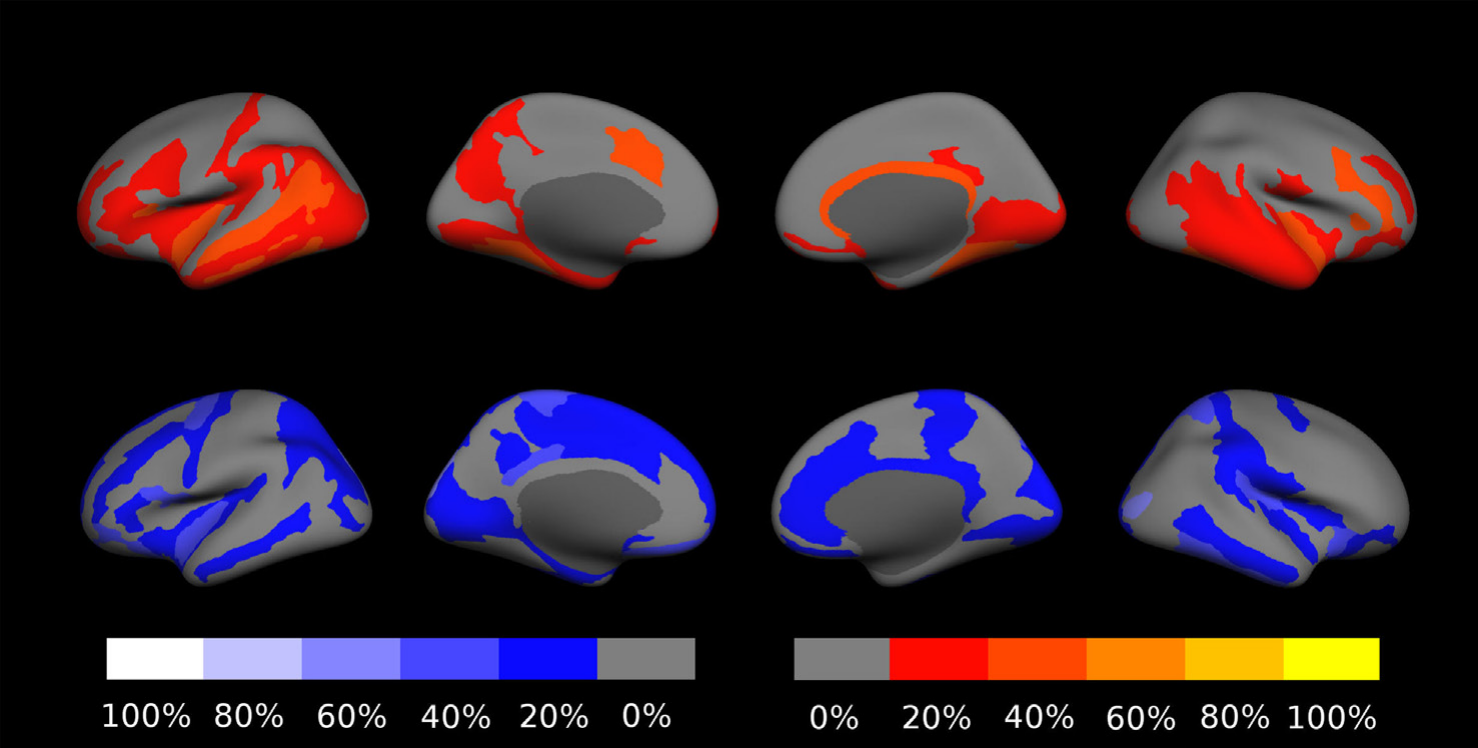
Percentage of patients showing excess linear change of cortical thickness during the follow-up period. The top row shows deviations toward stronger than expected increase or weaker than expected decrease on a red-to-yellow color scale. The bottom row shows deviations toward stronger decrease or weaker increase on a blue-to-white color scale. In Figure S18 in Supplementary Material, a similar result compilation is shown for changes in gray–white contrast.

**Figure 10:**
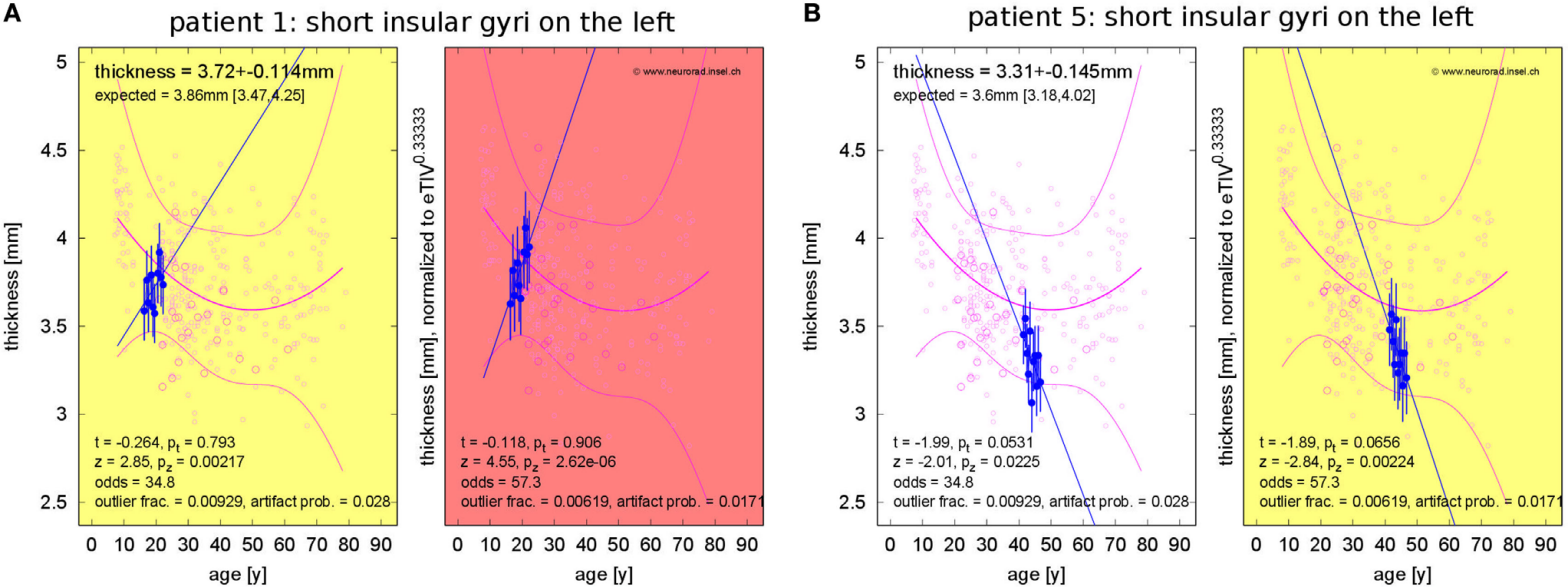
Progressive change of cortical thickness in the short insular gyri on the left. **(A)** Patient 1 shows thickness increase. **(B)** Patient 5 shows thickness loss.

Concerning changes of mean cortical thickness in individual patients, we found deviations from the expectation in both directions, see Figure 9. Weaker than expected thickness decrease (or even increase) was observed predominantly in the bilateral temporal lobes and insula. By contrast, stronger than expected thickness decrease was observed predominantly in the bilateral cingulate, parieto-occipital regions and in the pre- and postcentral gyri and sulci. In Figure 10, we demonstrate at the example of the short insular gyri on the left that the coexistence of thickness increase and decrease is not an artifact of our methodology. We found pronounced thickness increase in patient 1 (Figure 10A), whereas marked thickness decrease was detected in patient 5 (Figure 10B).

## Discussion

### Methodological Developments

Several studies using MR-based morphometry have indicated that GM atrophy in RR-MS emerges not only as a global but also rather regional process, affecting the temporal lobe, the pre- and postcentral gyrus, cingulate gyrus, basal ganglia, and the thalamus (44, 45). Long-term atrophy progression of GM was found associated with disability progression, whereas no effect was found for WM (18). To monitor longitudinal GM changes, we have employed a fully automatic pipeline for volumetric and morphometric evaluation of individual and follow-up high-resolution T1w MRI datasets (Figure 1) based on the free software packages FSL and FreeSurfer. We referenced our method to a large and extendable control group of currently *N* = 323 healthy subjects exhibiting a population-based distribution and tailored statistical post-processing accounting for age, sex, brain size, gray–white contrast, and technical parameters as potential confounders. The reliability of regional estimates of morphometric parameters was estimated from repeated MRI acquisitions with separation less than 2 years in a subset of 31 of our HCs (87 MRIs). Similarly, the HC outlier fraction observed in specific morphometric parameters and brain regions was used to estimate the corresponding artifact probability and, finally, the odds for valid versus erroneous observations. The larger these odds, the more the software’s estimates can be trusted.

All these quantifiers were integrated into a standardized result presentation (Figure 2) and can be made available to the expert to provide quantitative support for clinical MRI review. As some of the surface-based morphometric parameters (especially the surface area and curvatures of the highly folded cortical band) are barely amenable to visual inspection of MR slices, this complements the information available to the human expert.

The analysis pipeline underwent LOOCV in HCs. In a randomly selected subset of 34 HCs, it yielded low but significantly elevated anomaly rates (see Table 3). These deviations were most likely due to a slight violation of the implicit normality assumption by distributions with heavier tails. Testing a subset of 19 patient-matched HC datasets against the empirically expected anomaly rates, no major deviations were observed. By contrast, anomaly rates were significantly elevated by at least a factor of 2 in individual analysis of 19 follow-up MRI exams of two MS patients (see Table 3 and Table S4 in Supplementary Material).

### Use Case: Detecting Regional Atrophy in RR-MS

As a proof-of-concept, we applied the analysis to one use case, i.e., the longitudinal follow-up in patients with RR-MS (5 patients, 55 MRIs with a mean follow-up time of 5 years). Despite low to moderate disability in all five patients, the expanded disability status scale (EDSS) correlated with the time elapsed during the observation period in two of the patients (Table 1). Patient 3 had an extensive duration of disease and patient 4 had abnormal PVE in the first and last MRI of the follow-up series (Table 4). In all other patients, the PVE were normal in all MRIs of the follow-up series. Reduced cortical thickness in the first or last MRI of the follow-up series was found predominantly in the pre- and postcentral gyrus of both hemispheres and too small gray–white contrast was predominant in the left lateral temporal lobe (see Figures S14–S17 in Supplementary Material). Atrophy of deep GM structures was also observed in individual MRIs (see Figures S12 and S13 in Supplementary Material). These regional atrophy patterns are consistent with previous MS group studies (9–16) and have been assigned to cognitive impairment in MS. According to Steenwijk et al. (45) “cortical atrophy in multiple sclerosis occurs in a non-random manner and develops (at least partly) according to distinct anatomical patterns.” Thus, analysis of regional atrophy patterns need to be further explored as markers for clinical deterioration.

In addition to analysis of morphometric anomalies in volume segmentations and cortical parcellations at a single time point, the proposed pipeline was used to detect and quantify progression of regional atrophy by fitting a linear model and classifying whether the change rate deviated from the cross-sectional expectation (Figures 6–10). After normalization to eTIV, we found stronger than expected brain volume loss in 4/5 patients (Table 4). Cortical thickness change as a regional atrophy progression marker in individuals differed from the expectation in both directions, i.e., stronger than expected as well as weaker than expected thickness loss or even increase (Figures 9 and 10).

### Statistical and Methodological Considerations

Morphometric parameters vary systematically with age and sex (32–39). In addition, the performance of morphometry tools depends on image quality, which in turn is influenced by technical factors, such as MR scanner type and manufacturer, field strength, and acquisition sequence (39–41). In comparison between two groups of sizes N_sml_ and N_lrg_, these issues can be taken into account either by matching patients and HCs as exactly as possible or by introducing nuisance variables into the analysis. Unfortunately, at the clinically relevant limit where the smaller “group” is an individual patient or MRI dataset (N_sml_ = 1), the number of perfectly matched HCs available at most institutions is usually very small and application of nuisance parameters is mathematically hindered by the problem of inverting singular matrices.

A further difficulty with group discrimination is that statistical power crucially depends on the size of the smaller group and may, thus, be very weak in the extreme case where N_sml_ = 1. To deal with these problems, we analyzed residues after fitting low-order polynomial age trends to the morphometric parameters of the HCs (Figure 1) and restricted statistical testing to a suitable subset of HCs that matched the patient in all categorical characteristics (i.e., sex, MR type, and acquisition sequence). This constituted a compromise between minimization of potential sources of bias and maximizing the size N_lrg_ of the accessible control group—and thus the statistical power.

Our normative dataset contained neither HCs below 7 years nor subjects older than 79 years and had a gap between 13 and 17 years. It is important to note that application of the proposed statistical analysis of fit residues to patients with ages near or beyond the borders of the controls’ age range must be considered with extreme caution. The reason is that extrapolation of the polynomial fit to the age dependence of the included HCs rapidly loses reliability outside the age range covered by the normative dataset and in consequence might introduce large systematic errors in and beyond these border regions.

Direct comparison of longitudinal age trends derived from follow-up exams in the same patients with the cross-sectional age trend in the HC group must also be approached with caution. Our normative database contains repeated MRIs only from a relatively small number of subjects. As the temporal separation between repeated MRI exams was no longer than 2 years (i.e., small as compared to the whole age range of 7–79 years), we used these datasets to estimate measurement reliabilities rather than healthy aging trajectories. For future applications, one should consider building normative databases of follow-up MRIs in HCs.

In the current version of our pipeline, we omitted FreeSurfer’s time-consuming longitudinal analysis stream. Whereas, for vertex-wise analysis, it is expected that intra-subject image coregistration would increase reproducibility and accuracy of repeated measurements; due to the implicit averaging procedures, we do not expect great improvements in our current ROI-wise approach.

Besides volumes of total CSF, GM, and WM and volumes of segmentations, our pipeline primarily uses surface-based morphometric (SBA) parameters provided by the software FreeSurfer and reports region-wise averages. SBA has advantages, both over VBM analysis as well as over expert analysis. Classic VBM is limited to regional GM volumes and concentrations. Although non-standard VBM techniques are available to estimate cortical thickness (46, 47), their test–retest reliabilities were lower than for surface-based measurement in a population of dementia patients (48). Other morphometric parameters (cortical surface area and curvature) have not yet been assessed with VBM, neither are they easily accessible to expert MRI inspection. During slice-by-slice image analysis by an expert neuroradiologist, abnormal surface areas or curvatures in the highly curved cortical band remain undetectable to the reader.

## Conclusion

We propose a pipeline for automated morphometric analysis of individual and follow-up MRI exams. In the present study, the analysis pipeline, the outlier and artifact handling, and the statistical post-processing were developed and the feasibility was demonstrated. In future work, the tool needs to be evaluated in larger groups of MS patients and in patients with other pathologies. Importantly, the analysis concept presented here is by no means limited to FSL and FreeSurfer results as input data. An extension to input data generated by different software packages needs to be evaluated. Extension of our current segmentation- and parcelation-wise analysis to voxel- and vertex-wise analysis must also be addressed.

A first application to 55 datasets of five MS patients enabled us to detect critical regions of stable and progressing atrophy, which matched the predilection areas identified during group studies, i.e., focal thinning and loss of gray–white contrast in frontal, central, and temporal cortex regions as well as subcortical and cerebellar GM volume loss. Further studies with larger patient groups are necessary to confirm these preliminary findings. In addition, it remains to be investigated, whether individual atrophy progression or stability in these regions may be employed as surrogate markers for the efficiency of disease-modifying treatment in RR-MS.

Application to follow-up MRIs of patients and HCs proved high correlation of the feature vectors L of Eq. 5 within the same subject as well as within the RR-MS group. We, thus, envision that the feature vectors would be promising candidates for input to machine learning algorithms to classify not only subjects but also syndromes in MS and other pathologies such as epilepsy and dementia subtypes.

## Ethics Statement

This study was carried out in accordance with the recommendations of Kantonale Ethikkommission Bern with written informed consent from all subjects. All subjects gave written informed consent in accordance with the Declaration of Helsinki. The protocol was approved by the Kantonale Ethikkommission Bern (protocol 2016-02035).

## Author Contributions

CR, FW, and RW designed the study. CR and RM contributed software. CR and FA analyzed the imaging data. FA, FW, AS, and AC contributed clinical information. CR, FW, and RW wrote the manuscript. CR, FA, RM, FW, AS, AC, and RW reviewed and approved the final version of the manuscript.

## Conflict of Interest Statement

AC received research support from Genzyme and Novartis well as personal compensation for activities with Almirall, Bayer, Biogen, Genzyme, Merck, Novartis, Sanofi, and Teva. AS received personal compensation for activities with Novartis, Sanofi, and Almirall Hermal GmbH. None of the other authors has any conflict of interest to disclose.

## Footnotes

^1^http://fsl.fmrib.ox.ac.uk/fsl/fslwiki/.

^2^https://surfer.nmr.mgh.harvard.edu.

## Abbreviations

CIS, clinically isolated syndrome; CSF, cerebrospinal fluid; EDSS, expanded disability status scale; eTIV, estimated total intracranial volume; FDR, false discovery rate; FLAIR, fluid-attenuated inversion recovery; GM, gray matter; HC, healthy control; LOOCV, leave-one-out cross-validation; MRI, magnetic resonance imaging; MS, multiple sclerosis; NEDA, no evidence of disease activity; RR-MS, relapsing–remitting multiple sclerosis; SBA, surface-based analysis; T1w, T1-weighted; VBM, voxel-based morphometry; WM, white matter.
