# Supplementary material for: A Fully Automated Pipeline for Normative Atrophy in Patients with Neurodegenerative Disease
Source: Front Neurol. 2018 Jan 24;8:727. doi: 10.3389/fneur.2017.00727 (PMC5787548; doi:10.3389/fneur.2017.00727)
Supplement: Supplementary file 1 [file Presentation_1.PDF]

Supplementary Materials for

A fully automated pipeline for normative atrophy in patients with neurodegenerative disease

Christian Rummel[1+], Fabian Aschwanden[1], Richard McKinley[1], Franca Wagner[1], Anke Salmen[2], Andrew Chan[2], Roland Wiest[1]

[1]Support Center for Advanced Neuroimaging (SCAN), University Institute for Diagnostic and Interventional Neuroradiology, Inselspital Bern, University of Bern, Switzerland

[2]Department of Neurology, Inselspital Bern, University of Bern, Switzerland

[+]corresponding author:

Christian Rummel (PhD)

Support Center for Advanced Neuroimaging (SCAN)

University Institute for Diagnostic and Interventional Neuroradiology

Inselspital, 3010 Bern, Switzerland

Tel. 0041 31 6328038, Fax 0041 31 6324872, crummel@web.de

Supplementary methods

MR types and acquisition sequences

MR images of multiple sclerosis (MS) patients and healthy controls (HC) were acquired at the University Hospital Bern (Inselspital) on two 3T MR scanners (Magnetom Trio and Verio, Siemens, Erlangen, Germany). Every subject underwent a standardized T1-weighted MRI protocol including either a 3D MP-RAGE (Held *et al* 1995), an MDEFT (Deichmann *et al* 2004), an MP-RAGE according to the recommendations of the Alzheimer's Disease Neuroimaging Initiative (ADNI, Jack *et al* 2008) or an MP-RAGE optimized for grey–white contrast as recommended by van der Kouwe *et al* (2008). Images were acquired in sagittal acquisition and with 1 mm isotropic resolution. Detailed sequence parameters are summarized in Table S1.

	**MDEFT** (Deichmann *et al* 2004)	**MP-RAGE standard** (Held *et al* 1995)	**MP-RAGE** (ADNI, Jack *et al* 2008)	**MP-RAGE** (van der Kouve *et al* 2008)
**voxel size (mm)**	1.0*1.0*1.0	1.0*1.0*1.0	1.0*1.0*1.0	1.0*1.0*1.0
**field of view (mm)**	256*224	256*256	256*256	250*250
**slices**	176	160	160	160
**matrix**	256*224*176	256*256*160	256*256*160	250*250*160
**repetition time TR (ms)**	7.92	1500	2300	2530
**echo time TE (ms)**	2.48	2.01	2.98	3.37
**inversion time TI (ms)**	910	900	1100	1100
**flip angle (°)**	16	9	9	7
**fat saturation**	yes	no	no	no
**acquisition time (min)**	13'43”	3'30”	5'21”	10'49”

Table S1:
Basic parameters of the used MR acquisition sequences.

Healthy control data

The age and sex distribution of our normative database is provided in Figure S1. The database was generated by reusing anonymized data from HCs who had participated in earlier studies at the Inselspital. Only information on the subjects' age, sex, type of used MR scanner and acquisition sequence was kept. Detailed information on the studies, resulting publications and demographics of the subjects included is compiled in Table S2.

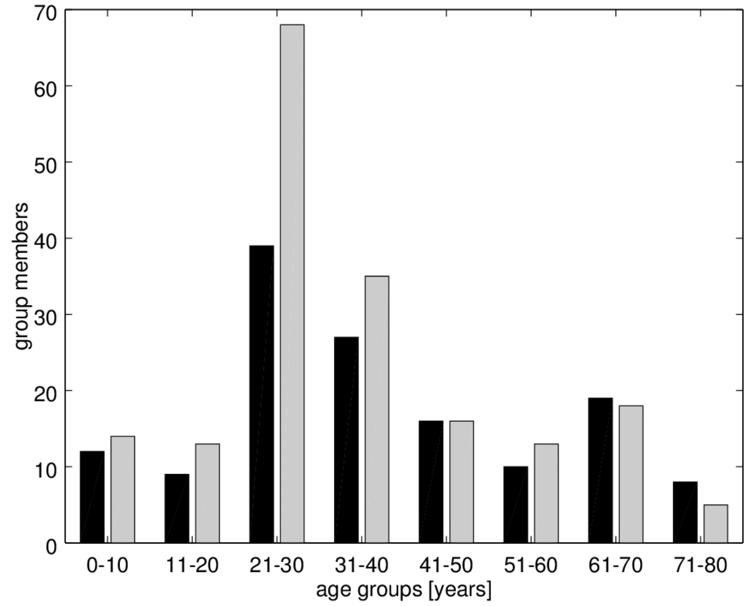

Figure S1:
Age and sex distribution of the 323 subjects included in the healthy control dataset (black: male, grey: female). Additional information is given in Table 2 of the main text.

Voxel-based volumetry (VBM) with FSL

The volumes of cerebro-spinal fluid (CSF), grey matter (GM) and white matter (WM) were estimated using the free software package FSL (`http://fsl.fmrib.ox.ac.uk/fsl/fslwiki/`, version 5.0, Smith *et al* 2004) developed at the Oxford Centre for Functional MRI of the Brain (FMRIB). First, the images were automatically skull-stripped using the Brain Extraction Tool (BET, version 2.1, Smith 2002) with robust brain center estimation (option `-R`) and underwent automated clean-up for eye and optic nerve contributions (option `-S`) as well as for bias field and neck (option

-B). The fractional intensity threshold (option -f) was set to 0.3. Using FMRIB's Automated Segmentation Tool (FAST, version 5.0.6, Zhang *et al* 2001) the brain voxels were then classified into three tissue types (CSF, GM and WM) with the sum of probabilities equal to one. Partial volume estimates (PVEs) for each tissue type were calculated by integrating the voxel-wise tissue type probabilities over the whole intracranial volume.

Volume segmentation with FreeSurfer

Volumes of subcortical GM, WM and CSF segmentations were estimated using the free software package FreeSurfer (`https://surfer.nmr.mgh.harvard.edu`, version 5.3.0) developed at the Laboratory for Computational Neuroimaging of the Athinoula A. Martinos Center for Biomedical Imaging. The procedures are described in detail in Fischl *et al* (2002,2004) and the segmentations include the hippocampus and the amygdala, the thalamus, the basal ganglia, the ventricles, the corpus callosum and the cerebellum.

surface-based analysis (SBA) with FreeSurfer

SBA was also performed with FreeSurfer. The technical details of these procedures have been described previously in Dale *et al* (1999) and Fischl *et al* (1999a,b). The processing includes automated tessellation of the GM–WM interface using vertices and simplices. Surfaces were deformed following intensity gradients to place the GM–WM and GM–CSF interfaces at the optimum location, where the greatest intensity change defines the transition to the other tissue class.

Nine morphometric parameters were evaluated at all vertex points of the surfaces and reported as averages over the cortex parcellations of the Desikan-Killiany atlas (Desikan *et al* 2006) with 34 parcellations per hemisphere and the Destrieux atlas (Destrieux *et al* 2010) with 74 parcellations per hemisphere:

- The surface area of a cortical parcellation was estimated by summing up (in native space) the face areas of all included simplices of the tessellated surface.

- Cortical thickness was calculated with sub-millimeter resolution as the shortest distance from the GM–WM interface to the GM–CSF interface at each vertex (Fischl *et al* 2000). Mean and standard deviation of the cortical thickness were evaluated for each parcellation.
- The parcellation-wise cortical GM volume was calculated as the sum of the vertex-wise products of cortical thickness and surface area.
- Curvature and folding measures were defined from the inverse radii of ellipsoids that approximate the surfaces locally. They can either be extrinsic properties of the surface embedded into 3D space or intrinsic properties of the surface itself (Pienaar *et al* 2008). The extrinsic mean curvature is given by the mean of the local minimal and maximal curvature (dimension 1/mm), whereas the intrinsic Gaussian curvature is the product of both (dimension $1/mm^2$).
- The grey–white contrast was estimated by extending the GM–WM interface 1 mm into the WM and 35% of the local cortical thickness into the GM according to Salat *et al* (2009). At voxel size 1 mm this procedure ensured that the target points were located in different voxels and the contrast was estimated as these voxels' intensity difference normalized to their intensity sum. It ranges between 0 for identical voxel intensities and 100% if one voxel has zero intensity.

Including asymmetry indices where appropriate, these morphometric parameters led to feature vectors of length 2,976 per MRI dataset, see Table S3.

Estimates of uncertainty

The measurement uncertainty σ_{meas} of a morphometric parameter X was estimated from the N_{rep} = 31 HCs with repeated MRI within two years (87 MRI datasets altogether):

$$\sigma_{meas} = \sqrt{\frac{\sum_{\nu=1}^{N_{rep}} \sum_{\mu=1}^{n(\nu)} \left(X_{\nu\mu} - \langle X_\nu \rangle \right)^2}{\sum_{\nu=1}^{N_{rep}} n(\nu) - 1}} \qquad (1)$$

where ν enumerates the subjects and μ each subject's n(ν) MRI datasets. The subject-specific average is given by:

$$\langle X_\nu \rangle = \frac{1}{n(\nu)} \sum_{\mu=1}^{n(\nu)} X_{\nu\mu} \qquad (2)$$

		global or on midline	left hemisphere	right hemisphere	asymmetry index
**partial volume estimates**	FSL	5			
**eTIV**	FreeSurfer	1			
**volume segmentations**	FreeSurfer	12	14	14	14
**Desikan-Killiany atlas**					
GM volume	FreeSurfer		34	34	34
surface area	FreeSurfer		34	34	34
mean thickness	FreeSurfer		34	34	34
standard deviation of thickness	FreeSurfer		34	34	34
mean curvature	FreeSurfer		34	34	34
Gaussian curvature	FreeSurfer		34	34	34
curvature index	FreeSurfer		34	34	34
folding index	FreeSurfer		34	34	34
grey-white contrast	FreeSurfer		34	34	34
**Desikan-Killiany atlas**					
GM volume	FreeSurfer		74	74	74
surface area	FreeSurfer		74	74	74
mean thickness	FreeSurfer		74	74	74
standard deviation of thickness	FreeSurfer		74	74	74
mean curvature	FreeSurfer		74	74	74
Gaussian curvature	FreeSurfer		74	74	74
curvature index	FreeSurfer		74	74	74
folding index	FreeSurfer		74	74	74
grey-white contrast	FreeSurfer		74	74	74
sum					2976

Table S3:
Composition of the feature vectors by morphometric parameters.

Supplementary results

Mutual dependence between morphometric parameters

To explore mutual dependencies among the nine morphometric parameters for cortical parcellations we evaluated their Pearson correlation matrix in all 216 regions of interest (on both hemispheres – 34 from the Desikan-Killiany atlas and 74 from the Destrieux atlas) using the full set of 323 HCs. Results are displayed in Figure S2. To account for the global effect of brain size, all parameters except the grey–white contrast were isometrically scaled to the mean estimated total intracranial volume ($eTIV^n$) before correlation analysis as described in detail in the main text. Likewise, the grey–white contrast was normalized to the same regional mean across MR scanners and acquisition sequences.

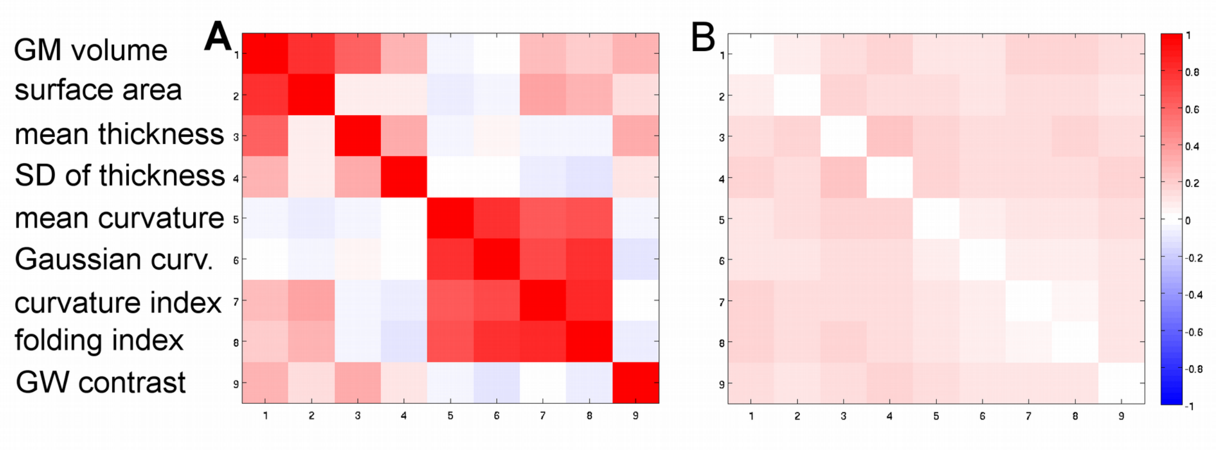

Figure S2:
Mean (left) and standard deviation (right) of Pearson's parcellation-wise correlation coefficients between different morphometric parameters calculated from all healthy controls. Before analyzing correlation, all parameters were normalized to $eTIV^n$.

Among the surface-based morphometric parameters, the mean cortical thickness and the cortical surface area are measures of the vertical and horizontal organization of the neocortex, respectively. Cortical thickness may decrease when neurons degrade or shrink and cortical surface area may increase when sulci become deeper due to changes in the underlying WM fibers. Our finding of low Pearson correlation between these parameters in HCs is consistent with the findings of a study by Winkler *et al* (2010) showing genetic and phenotypical independence of thickness and surface area. The grey–white contrast was

found to be anti-correlated with all four curvature parameters. This is because, for weaker contrast, the grey–white and the pial surfaces become less defined and curvatures typically increase. This uncertainty affects curvature parameters (defined as inverse radii) more than other surface-based parameters.

Distributions of polynomial age fits and outlier fractions

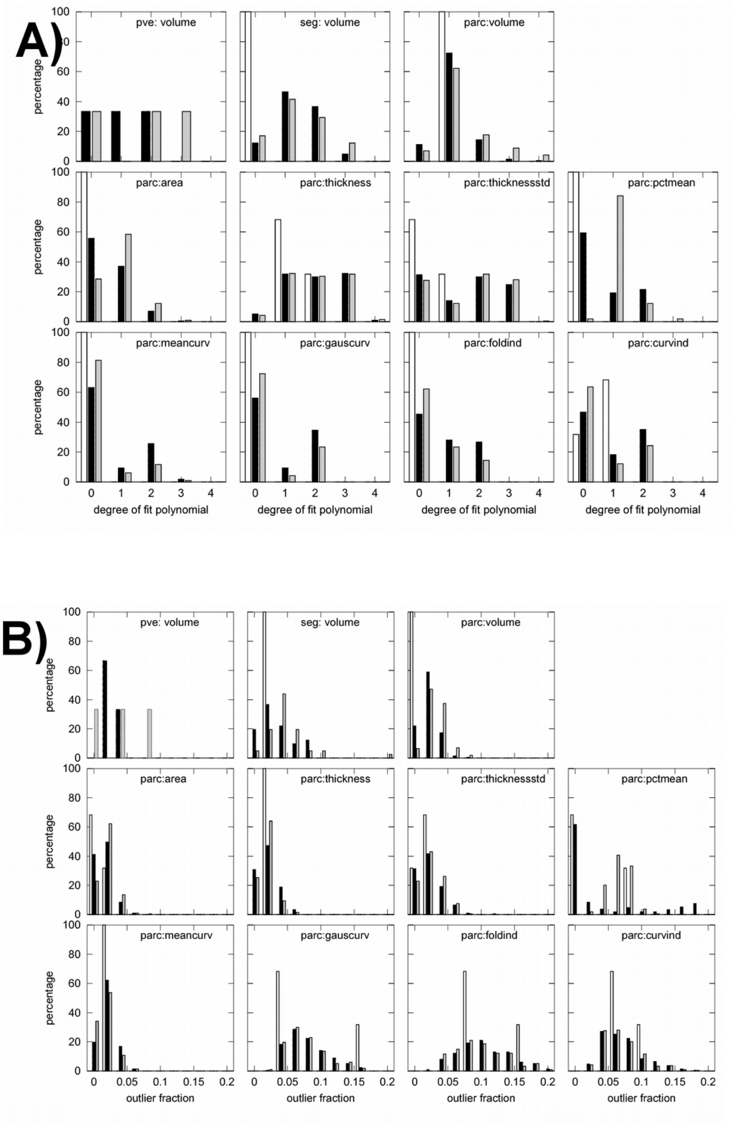

Figure S3:
A) Degree distributions of the best polynomial age fits to all morphometric parameters measured in the healthy controls. Degrees d > 4 never occurred although the maximally allowed degree was d_{max} = 16.
B) Distributions of outlier fractions of morphometric parameters in healthy controls.
White, asymmetry indices; black, raw parameters on both hemispheres; grey, parameters after normalization to $eTIV^n$ (areas, thicknesses, volumes and curvatures) or scanner and sequence (grey–white contrast).

Spatial distribution of odds for valid versus erroneous (i.e. artifact-corrupted) morphometric parameter estimates

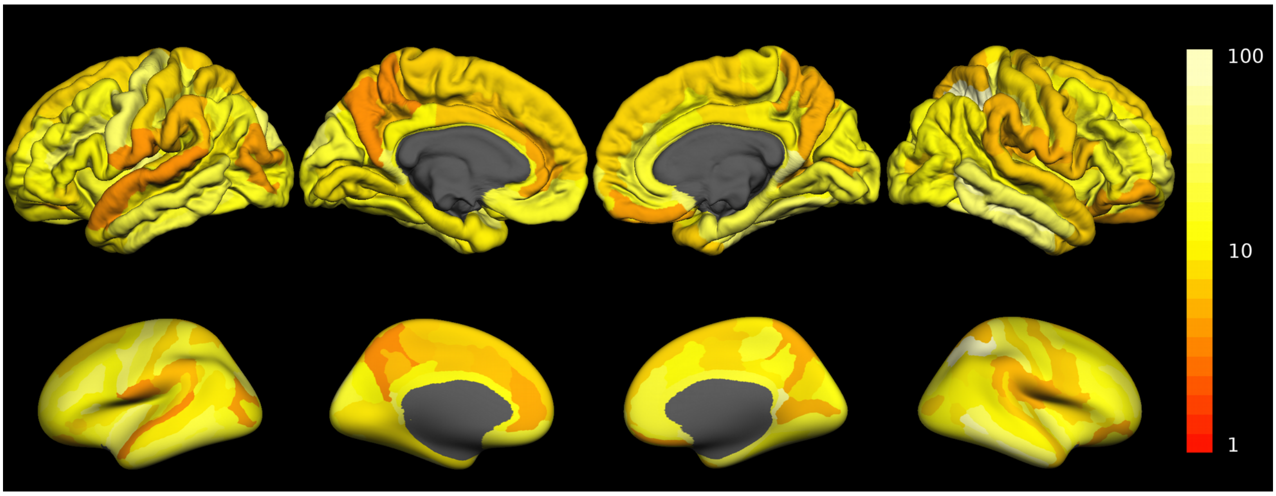

Figure S4:
Spatial distribution of the odds for valid versus erroneous measurement of the eTIV normalized **cortical grey matter volume**. The figure arrangement is the same as in Figure 4 of the main text.

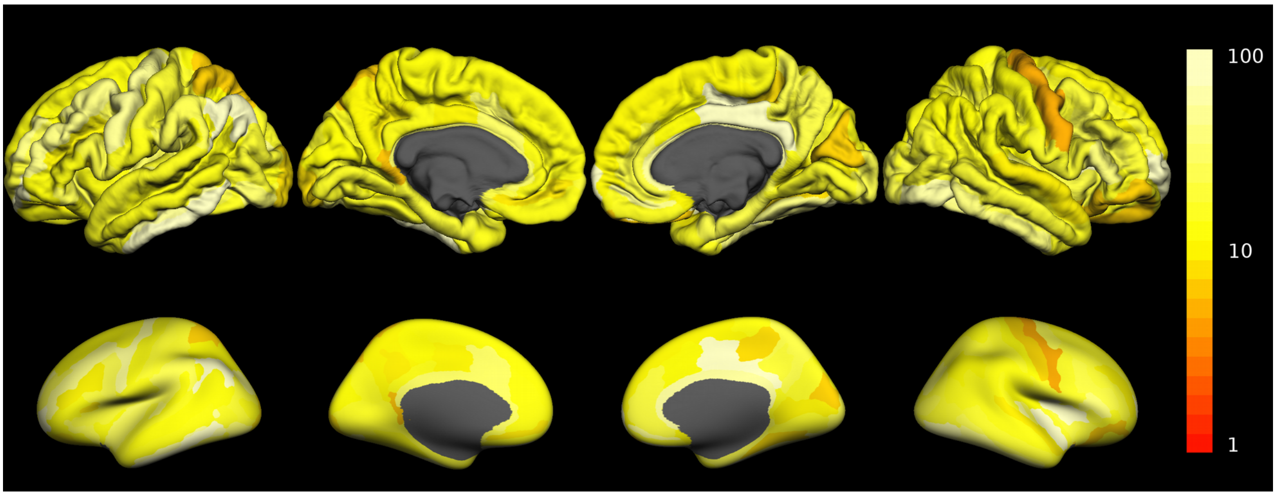

Figure S5:
Spatial distribution of the odds for valid versus erroneous measurement of the eTIV normalized **cortical surface area**. The figure arrangement is the same as in Figure 4 of the main text.

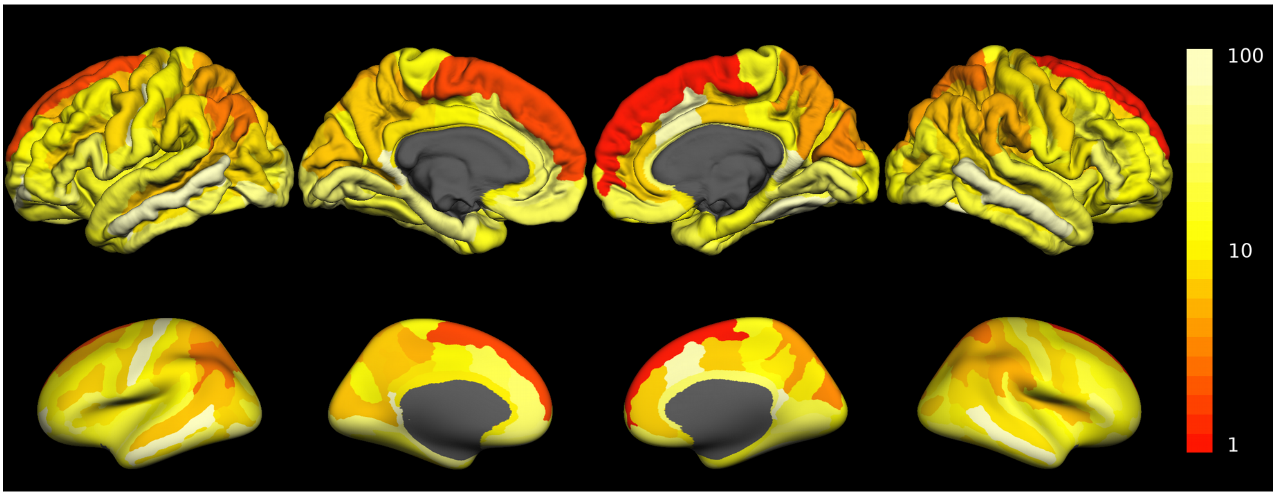

Figure S6:
Spatial distribution of the odds for valid versus erroneous measurement of the eTIV normalized **standard deviation of the cortical thickness**. The figure arrangement is the same as in Figure 4 of the main text.

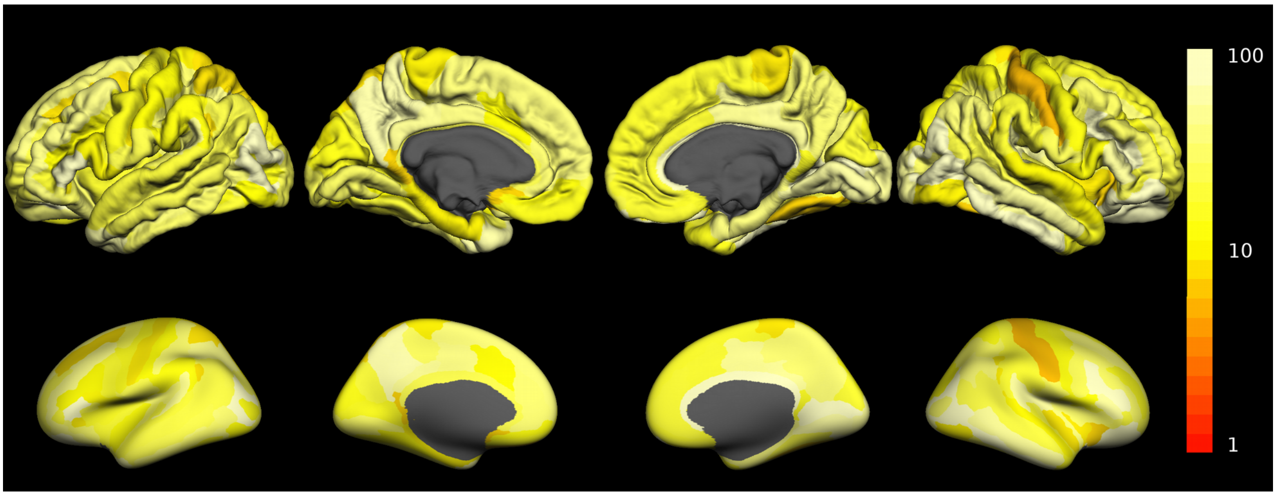

Figure S7:
Spatial distribution of the odds for valid versus erroneous measurement of the eTIV normalized **mean cortical curvature**. The figure arrangement is the same as in Figure 4 of the main text.

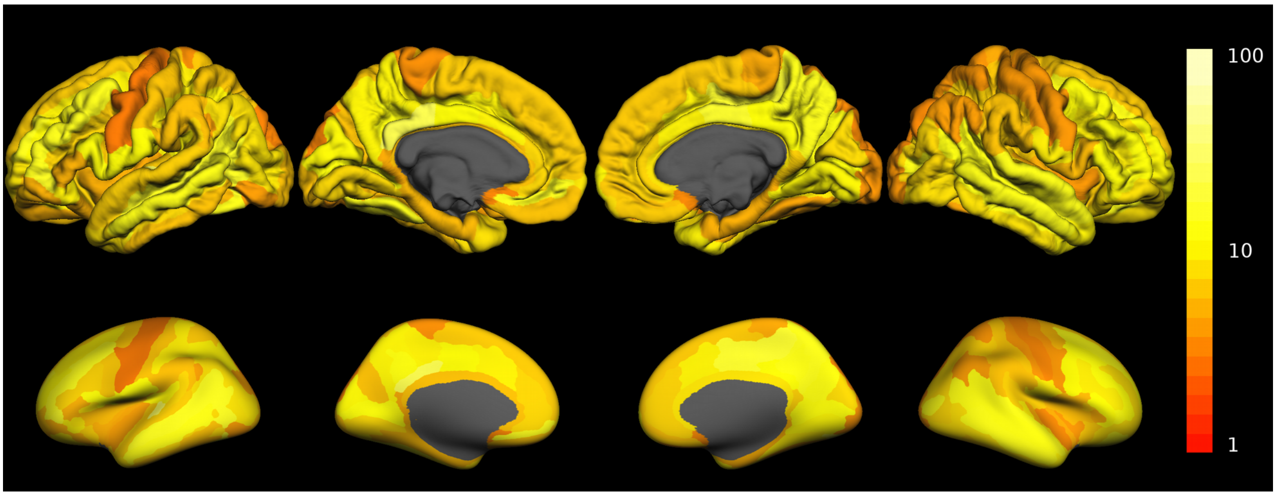

Figure S8:
Spatial distribution of the odds for valid versus erroneous measurement of the eTIV normalized **Gaussian curvature** of the cortex. The figure arrangement is the same as in Figure 4 of the main text.

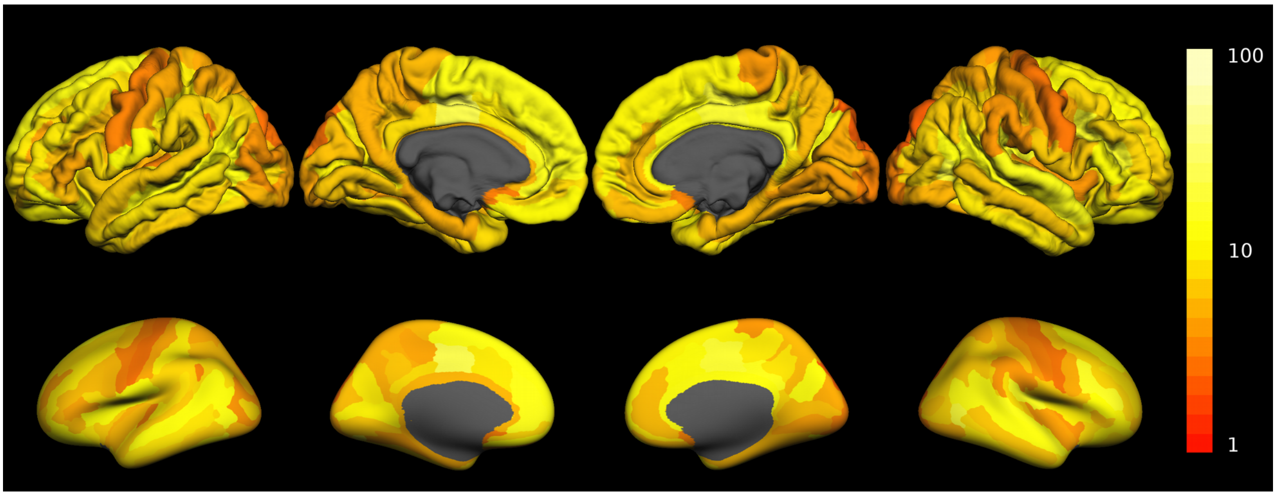

Figure S9:
Spatial distribution of the odds for valid versus erroneous measurement of the eTIV normalized **curvature index** of the cortex. The figure arrangement is the same as in Figure 4 of the main text.

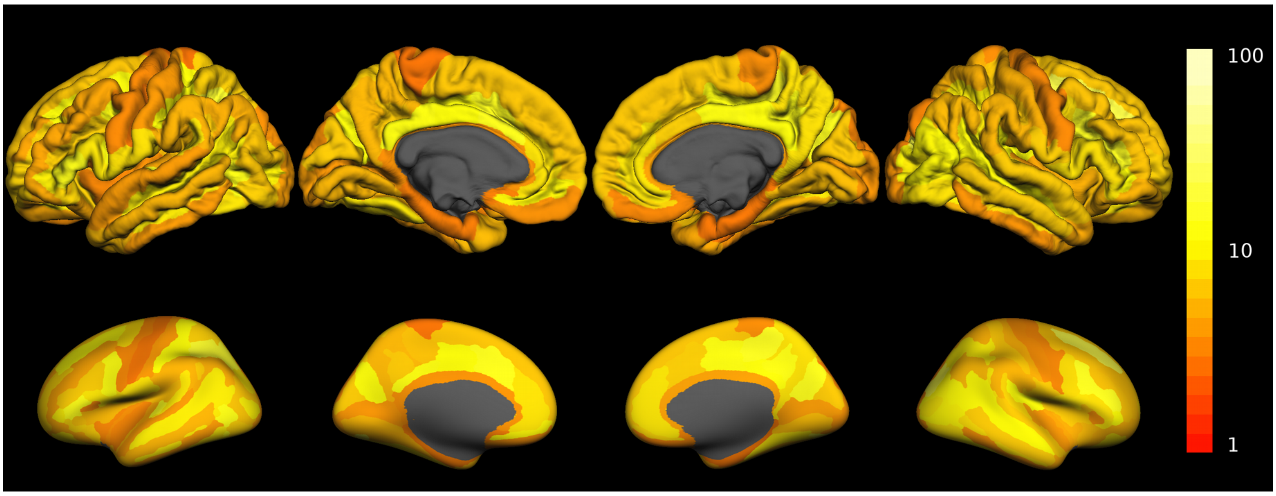

Figure S10:
Spatial distribution of the odds for valid versus erroneous measurement of the eTIV normalized **folding index** of the cortex. The figure arrangement is the same as in Figure 4 of the main text.

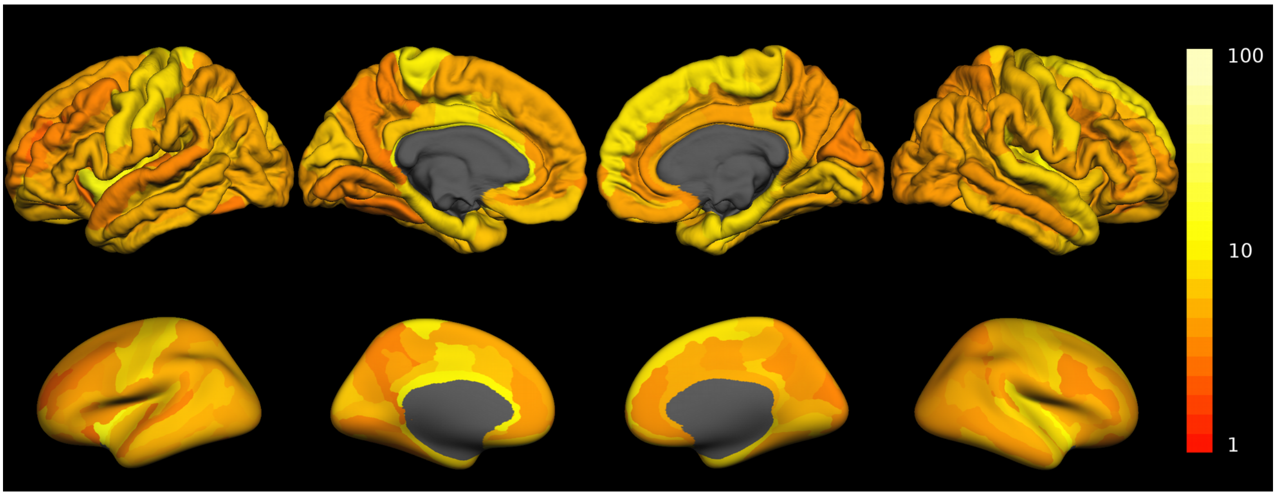

Figure S11:
Spatial distribution of the odds for valid versus erroneous measurement of the scanner and sequence normalized **cortical grey–white contrast**. The figure arrangement is the same as in Figure 4 of the main text.

Volume segmentation anomalies in the MS patient group

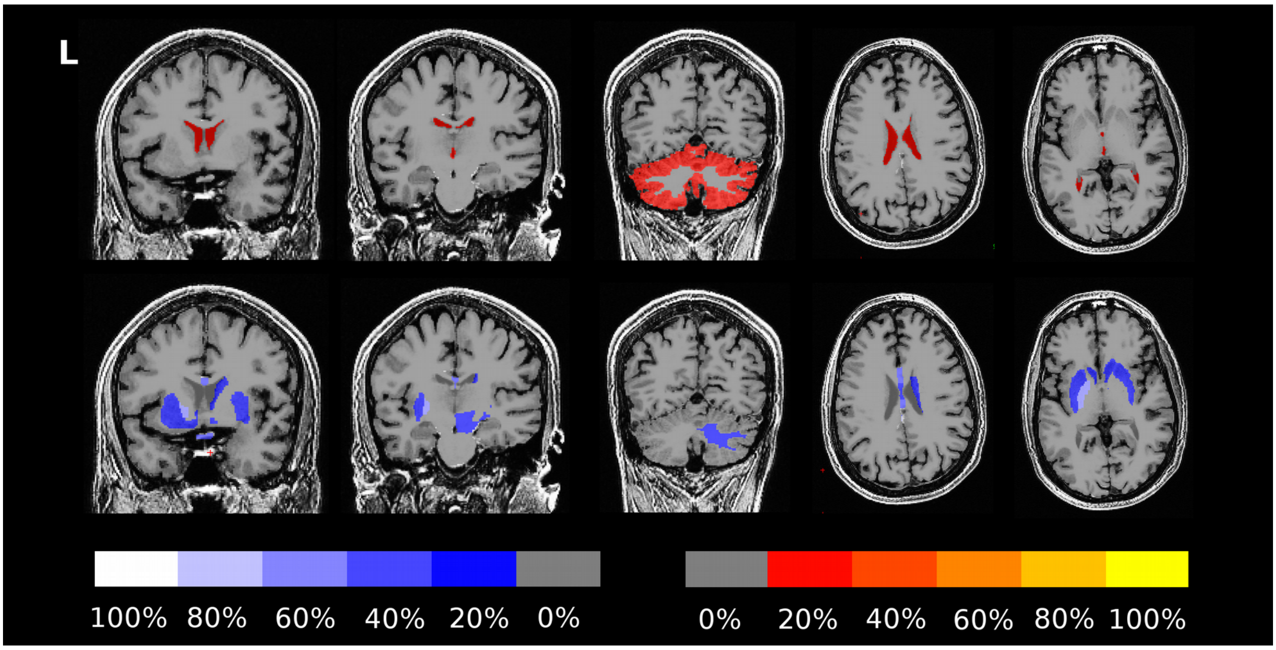

Figure S12:
Percentage of patients with significant deviations of eTIV normalized **volume segmentations** from the age and sex corrected expectation at the **first MRI** of the follow-up series. The figure arrangement is the same as in Figure 7 of the main text, where the volume change rate is displayed.

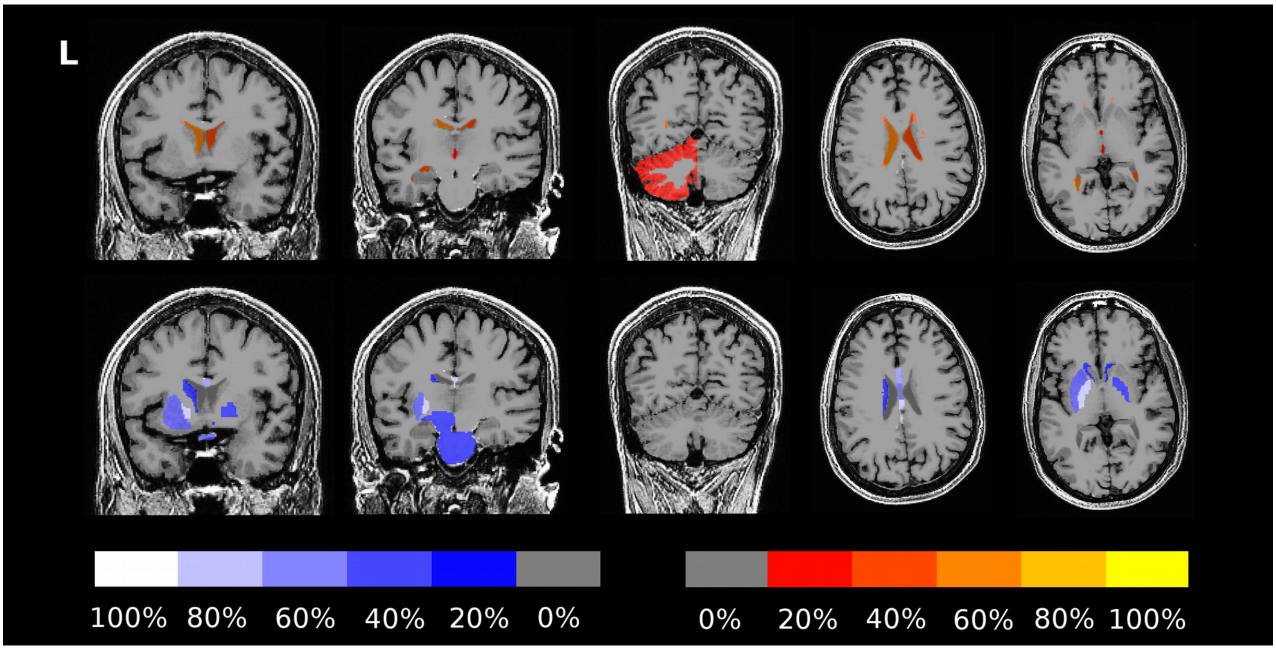

Figure S13:
Percentage of patients with significant deviations of eTIV normalized **volume segmentations** from the age and sex corrected expectation at the **last MRI** of the follow-up series. The figure arrangement is the same as in Figure 7 of the main text, where the volume change rate is displayed.

Cortical thickness anomalies in the MS patient group

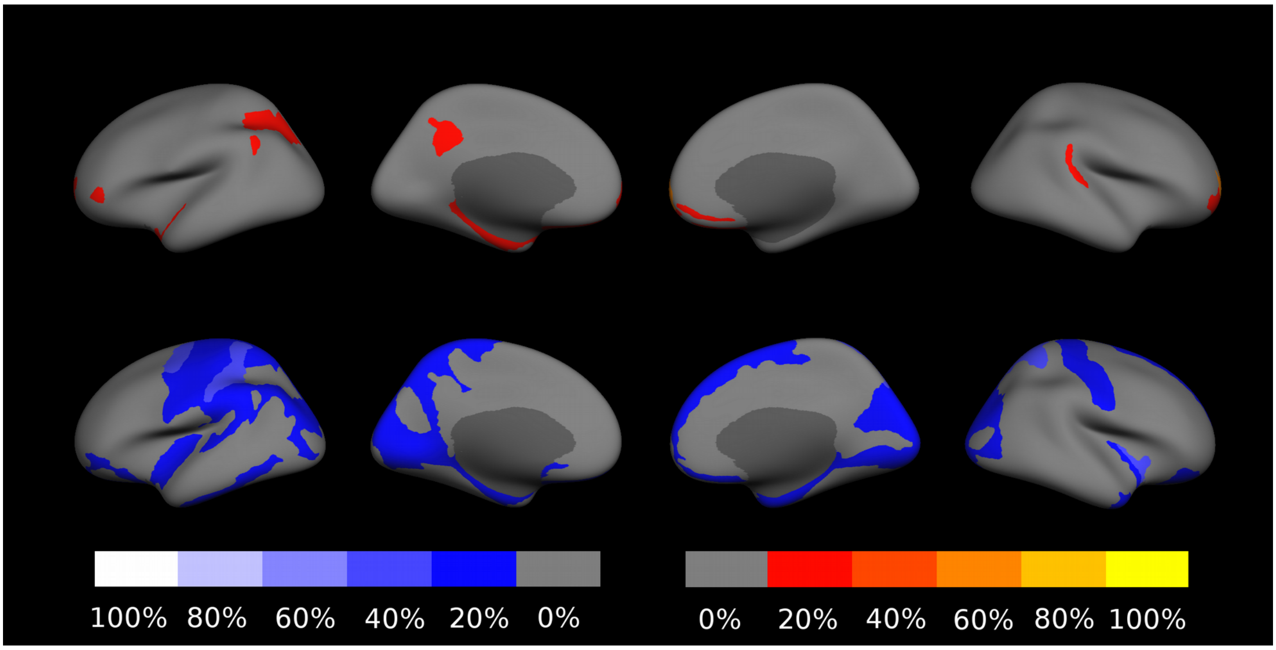

Figure S14:
Percentage of patients with significant deviations of eTIV normalized **mean cortical thickness** from the age and sex corrected expectation at the **first MRI** of the follow-up series. The figure arrangement is the same as in Figure 9 of the main text, where the thickness change rate is displayed.

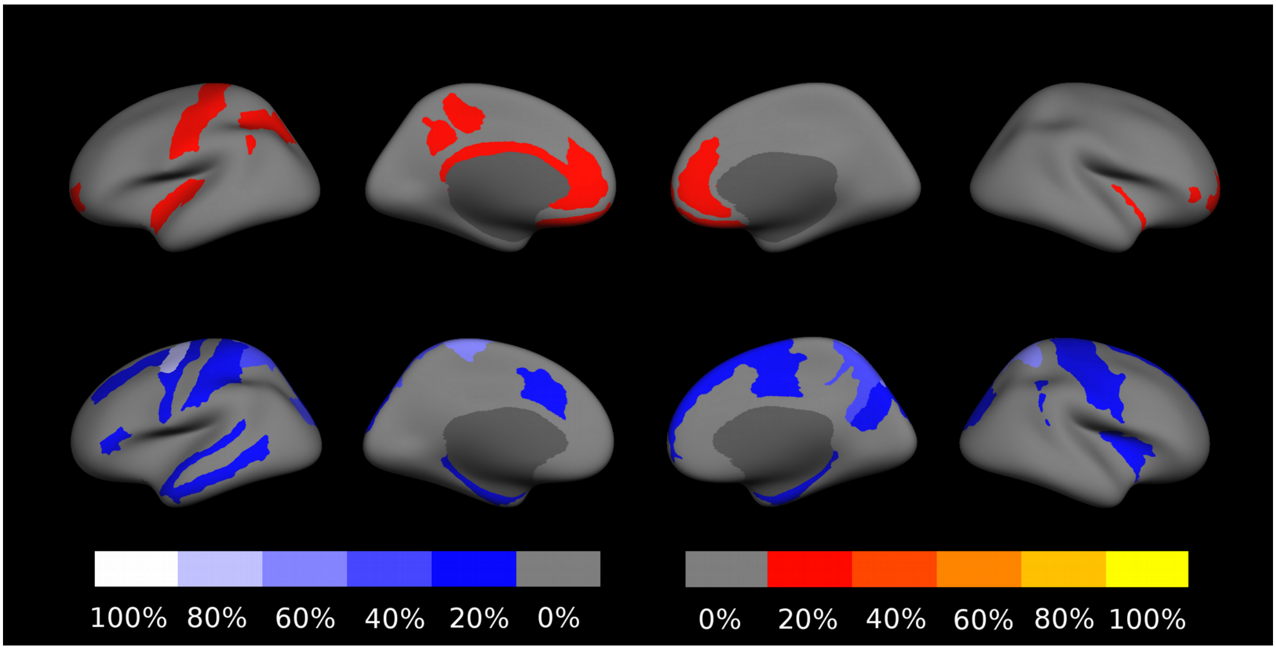

Figure S15:
Percentage of patients with significant deviations of eTIV normalized **mean cortical thickness** from the age and sex corrected expectation at the **last MRI** of the follow-up series. The figure arrangement is the same as in Figure 9 of the main text, where the thickness change rate is displayed.

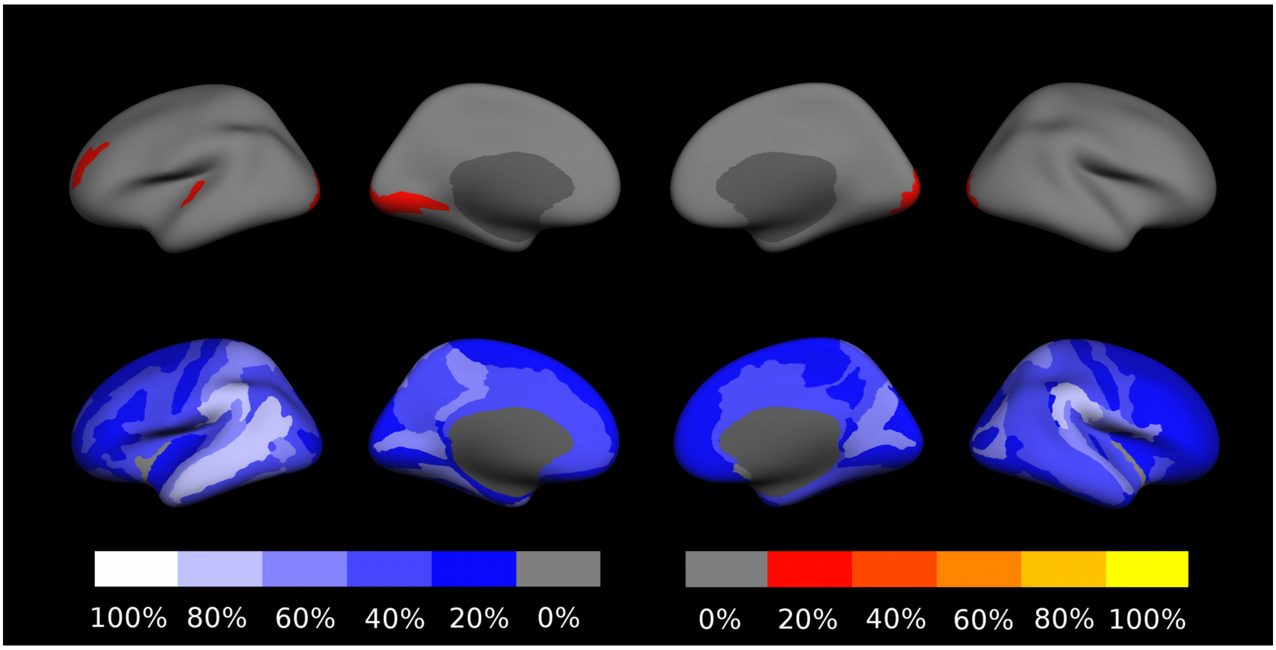

Figure S16:
Percentage of patients with significant deviations of scanner-sequence normalized **grey-white contrast** from the age and sex corrected expectation at the **first MRI** of the follow-up series. The figure arrangement is the same as in Figure 9 of the main text.

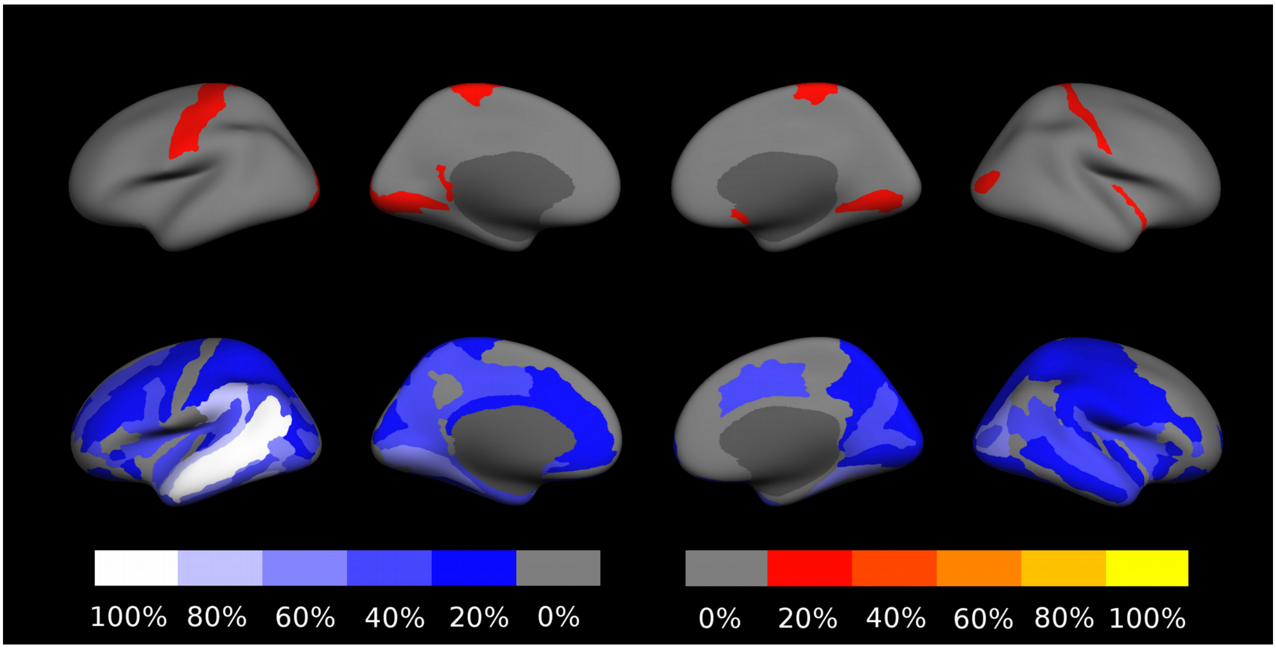

Figure S17:
Percentage of patients with significant deviations of scanner-sequence normalized **grey-white contrast** from the age and sex corrected expectation at the **last MRI** of the follow-up series. The figure arrangement is the same as in Figure 9 of the main text.

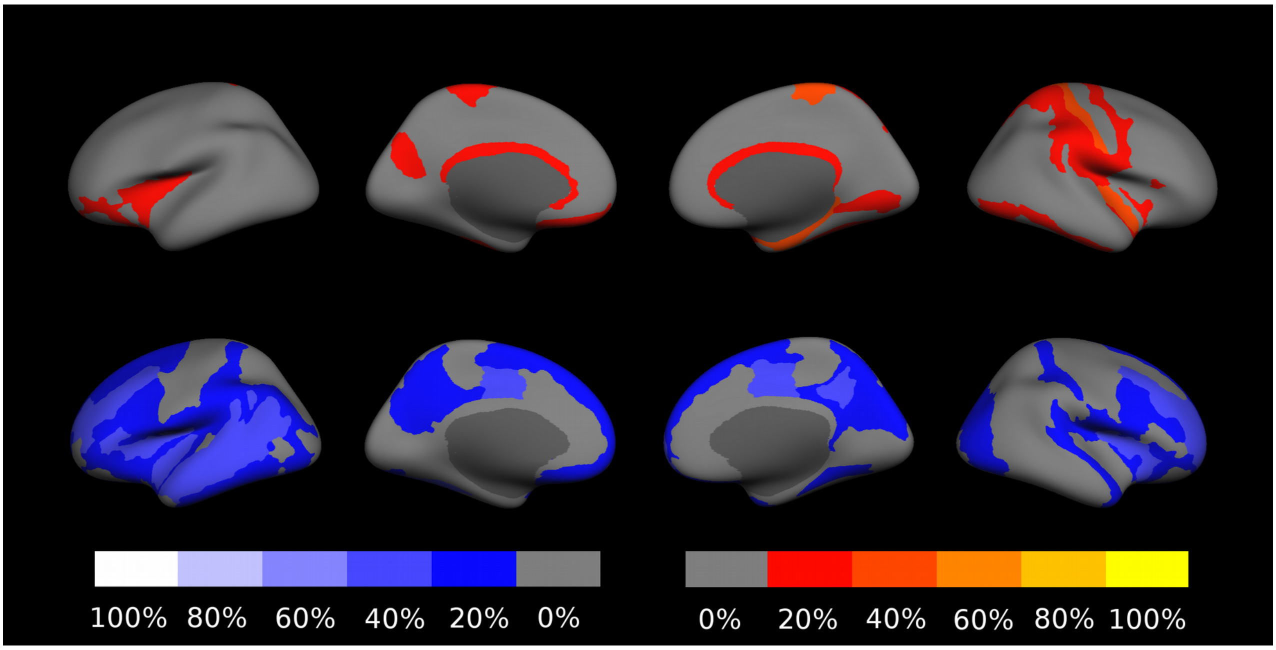

Figure S18:
Percentage of patients with significant deviations of the **change rate** of scanner-sequence normalized **grey-white contrast** from the age and sex corrected expectation. The figure arrangement is the same as in Figure 9 of the main text.

Table S2

reference	MR scanner	acquisition sequence	remarks	number in publication	number acquired	number included in database	male / female included in database	age included in database
Schweizer S. Bestimmung der kortikalen Dicke des Gehirns bei Probanden mittels Magnettomographie, master thesis, University of Applied Sciences Bern (2011).	MR 5 and MR 6	MDEFT, MPR (standard, ADNI and van der Kouwe)	master's thesis for exploration of best suited sequence for morphometric analysis; master's thesis, unpublished	15	34	34	4 / 30	27.6 ± 5.4 (21 – 40)
Mordasini L et al. Chronic pelvic pain syndrome: Back to the brain. J. Urol. 188, 2233-2237 (2012).	MR 6	MDEFT	-	20	21	21	21 / 0	38.0 ± 16.6 (22 – 72)
Abela E et al., Lesions to Primary Sensory and Posterior Parietal Cortices Impair Recovery from Hand Paresis after Stroke. PloS ONE 7, e31275 (2012)	MR 6	MDEFT	-	22	22	19	7 / 12	66.6 ± 6.0 (49 – 73)
Rummel C et al. Time course based artifact identification for independent components of resting state fMRI. Front. Hum. Neurosci. 7, 214 (2013).	MR 5	MDEFT and MPR van der Kouwe	two structural scans in each participant, both included in the normative data base, only MDEFT evaluated in the earlier paper	36	72	65	14 / 51	34.2 ± 10.4 (21 – 61)
Wiest R et al. Widespread grey matter changes and hemodynamic correlates to interictal epileptiform discharges in pharmacoresistant mesial temporal epilepsy. J. Neurol. 260, 1601-1610 (2013).	MR 6	standard MPR	-	10	39	38	17 / 21	35.3 ± 7.7 (23 – 66)
Mürner-Lavanchy I et al. Delay of cortical thinning in very preterm born children. Early Hum. Dev. 90, 443-450 (2014).	MR 5	MPR ADNI	-	30	42	42	21 / 21	9.6 ± 1.6 (7.2 – 12.9)
Vanbellingen T et al., Left posterior parietal theta burst stimulation affects gestural imitation regardless of semantic content. Clin. Neurophysiol. 125, 457–462 (2014)	MR 6	standard MPR	-	12	12	7	3 / 4	59.3 ± 12.1 (34 – 68)

Kottke R et al. Morphological Brain Changes after Climbing to Extreme Altitudes—A Prospective Cohort Study. PloS ONE 0141097 (2015).	MR 5	MDEFT	pre- and post-expedition MRI in all participants, only pre-expedition MRIs included in the normative data base	40	41	40	22 / 18	45.4 ± 12.1 (24 – 69)
Wiest R et al. Model-based magnetization transfer imaging to segregate HD huntigton's disease patients, asymptomatic gene carries and healthy controls. (under revision)	MR 5	MPR ADNI	-	10	10	5	5 / 0	50.6 ± 7.5 (46 – 64)
-	MR 6	MDEFT	Distinct activation patterns within the motor network in excentric vs. concentric movements (ongoing)	-	53	39	21 / 18	46.1 ± 21.9 (18 – 79)
-	MR 6	standard MPR	Processing of aesthetic perception in the architect's brain (ongoing)	-	13	13	7 / 6	27.8 ± 9.9 (17 – 51)
**sum**				**195**	**359**	**323**	**142 / 181**	**35.9 ± 18.0 (7 – 79)**

Table S2:
Additional information on earlier studies. Healthy controls acquired for these studies were reused in the normative database of the present study in anonymized form.

Table S4A

		test count	LOOCV: 34 random HCs p < 0.01 p_uncorr	p_FDR	p < 0.05 p_uncorr	p_FDR
**PVE**						
raw	count	102	0	0	2	0
	percentage		0.00%	0.00%	1.96%	0.00%
	p_bino (nominal)		0.359	n.a.	0.110	n.a.
	p_bino (empirical)		--	--	--	--
eTIV normalized	count	102	0	0	2	0
	percentage		0.00%	0.00%	1.96%	0.00%
	p_bino (nominal)		0.359	n.a.	0.110	n.a.
	p_bino (empirical)		--	--	--	--
**volume segmentations**						
raw	count	1360	29	6	100	8
	percentage		2.13%	0.44%	7.35%	0.59%
	p_bino (nominal)		**$<10^{-5}$**	n.a.	**$<10^{-4}$**	n.a.
	p_bino (empirical)		--	--	--	--
eTIV normalized	count	1360	30	6	101	7
	percentage		2.21%	0.44%	7.43%	0.51%
	p_bino (nominal)		**$<10^{-4}$**	n.a.	**$<10^{-4}$**	n.a.
	p_bino (empirical)		--	--	--	--
asymmetry	count	476	13	1	33	2
	percentage		2.73%	0.21%	6.93%	0.42%
	p_bino (nominal)		0.001	n.a.	0.039	n.a.
	p_bino (empirical)		--	--	--	--
**cortical GM volume**						
raw	count	7344	100	7	463	18
	percentage		1.36%	0.10%	6.30%	0.25%
	p_bino (nominal)		0.002	n.a.	**$<10^{-6}$**	n.a.
	p_bino (empirical)		--	--	--	--
eTIV normalized	count	7344	102	7	471	18

		percentage		1.39%	0.10%	6.41%	0.25%
		p_bino (nominal)		$<10^{-3}$	n.a.	**$<10^{-7}$**	n.a.
		p_bino (empirical)		--	--	--	--
	asymmetry	count	3672	32	0	236	0
		percentage		0.87%	0.00%	6.43%	0.00%
		p_bino (nominal)		0.246	n.a.	**$<10^{-4}$**	n.a.
		p_bino (empirical)		--	--	--	--
**surface area**							
	raw	count	7344	84	6	469	11
		percentage		1.14%	0.08%	6.39%	0.15%
		p_bino (nominal)		0.120	n.a.	**$<10^{-7}$**	n.a.
		p_bino (empirical)		--	--	--	--
	eTIV normalized	count	7344	91	6	492	11
		percentage		1.24%	0.08%	6.70%	0.15%
		p_bino (nominal)		0.026	n.a.	**$<10^{-9}$**	n.a.
		p_bino (empirical)		--	--	--	--
	asymmetry	count	3672	56	2	246	4
		percentage		1.53%	0.05%	6.70%	0.11%
		p_bino (nominal)		0.002	n.a.	**$<10^{-5}$**	n.a.
		p_bino (empirical)		--	--	--	--
**mean thickness**							
	raw	count	7344	111	4	371	19
		percentage		1.51%	0.05%	5.05%	0.26%
		p_bino (nominal)		**$<10^{-4}$**	n.a.	0.427	n.a.
		p_bino (empirical)		--	--	--	--
	eTIV normalized	count	7344	114	4	505	11
		percentage		1.55%	0.05%	6.88%	0.15%
		p_bino (nominal)		$<10^{-5}$	n.a.	**$<10^{-11}$**	n.a.
		p_bino (empirical)		--	--	--	--
	asymmetry	count	3672	37	0	212	2
		percentage		1.01%	0.00%	5.77%	0.05%
		p_bino (nominal)		0.504	n.a.	0.019	n.a.

		p_bino (empirical)		--	--	--	--
**SD of thickness**							
	raw	count	7344	92	8	371	18
		percentage		1.25%	0.11%	5.05%	0.25%
		p_bino (nominal)		0.020	n.a.	0.427	n.a.
		p_bino (empirical)		--	--	--	--
	eTIV normalized	count	7344	97	8	391	19
		percentage		1.32%	0.11%	5.32%	0.26%
		p_bino (nominal)		0.005	n.a.	0.107	n.a.
		p_bino (empirical)		--	--	--	--
	asymmetry	count	3672	49	0	239	2
		percentage		1.33%	0.00%	6.51%	0.05%
		p_bino (nominal)		0.030	n.a.	**$<10^{-4}$**	n.a.
		p_bino (empirical)		--	--	--	--
**mean curvature**							
	raw	count	7344	75	11	377	18
		percentage		1.02%	0.15%	5.13%	0.25%
		p_bino (nominal)		0.443	n.a.	0.307	n.a.
		p_bino (empirical)		--	--	--	--
	eTIV normalized	count	7344	75	11	384	18
		percentage		1.02%	0.15%	5.23%	0.25%
		p_bino (nominal)		0.443	n.a.	0.191	n.a.
		p_bino (empirical)		--	--	--	--
	asymmetry	count	3672	60	9	273	12
		percentage		1.63%	0.25%	7.43%	0.33%
		p_bino (nominal)		$<10^{-3}$	n.a.	**$<10^{-9}$**	n.a.
		p_bino (empirical)		--	--	--	--
**Gaussian curvature**							
	raw	count	7344	186	102	308	131
		percentage		2.53%	1.39%	4.19%	1.78%
		p_bino (nominal)		**0**	n.a.	$<10^{-3}$	n.a.

		p_bino (empirical)		--	--	--	--
	eTIV normalized	count	7344	190	106	314	135
		percentage		2.59%	1.44%	4.28%	1.84%
		p_bino (nominal)		**0**	n.a.	0.002	n.a.
		p_bino (empirical)		--	--	--	--
	asymmetry	count	3672	120	14	270	33
		percentage		3.27%	0.38%	7.35%	0.90%
		p_bino (nominal)		**0**	n.a.	**$<10^{-9}$**	n.a.
		p_bino (empirical)		--	--	--	--
**folding index**							
	raw	count	7344	219	126	353	148
		percentage		2.98%	1.72%	4.81%	2.02%
		p_bino (nominal)		**0**	n.a.	0.233	n.a.
		p_bino (empirical)		--	--	--	--
	eTIV normalized	count	7344	222	128	364	150
		percentage		3.02%	1.74%	4.96%	2.04%
		p_bino (nominal)		**0**	n.a.	0.446	n.a.
		p_bino (empirical)		--	--	--	--
	asymmetry	count	3672	93	16	287	26
		percentage		2.53%	0.44%	7.82%	0.71%
		p_bino (nominal)		**$<10^{-14}$**	n.a.	**$<10^{-12}$**	n.a.
		p_bino (empirical)		--	--	--	--
**curvature index**							
	raw	count	7344	178	73	323	93
		percentage		2.42%	0.99%	4.40%	1.27%
		p_bino (nominal)		**0.000**	n.a.	0.009	n.a.
		p_bino (empirical)		--	--	--	--
	eTIV normalized	count	7344	181	73	330	93
		percentage		2.46%	0.99%	4.49%	1.27%
		p_bino (nominal)		**0.000**	n.a.	0.023	n.a.
		p_bino (empirical)		--	--	--	--
	asymmetry	count	3672	77	10	242	28

		percentage		2.10%	0.27%	6.59%	0.76%
		p_bino (nominal)		**$<10^{-8}$**	n.a.	**$<10^{-4}$**	n.a.
		p_bino (empirical)		--	--	--	--
**grey-white contrast**							
	raw	count	7344	14	0	194	0
		percentage		0.19%	0.00%	2.64%	0.00%
		p_bino (nominal)		***$<10^{-16}$***	n.a.	**$<10^{-23}$**	n.a.
		p_bino (empirical)		--	--	--	--
	scanner/sequence corrected	count	7344	14	0	194	0
		percentage		0.19%	0.00%	2.64%	0.00%
		p_bino (nominal)		***$<10^{-16}$***	n.a.	**$<10^{-23}$**	n.a.
		p_bino (empirical)		--	--	--	--
	asymmetry	count	3672	45	4	206	5
		percentage		1.23%	0.11%	5.61%	0.14%
		p_bino (nominal)		**0.101**	n.a.	0.051	n.a.
		p_bino (empirical)		--	--	--	--

Table S4A:
Parameter-specific anomalies detected in the randomly selected LOOCV test set at two significance levels $\alpha = 0.01$ and $\alpha = 0.05$. The 34 MRI datasets were tested against the expected error rate α (binomial tests). The table arrangement corresponds exactly to that of Table 3 of the main text.
Abbreviations: FDR, false discovery rate; LOOCV, leave-one-out cross-validation; n.a., not applicable.

Table S4B

				LOOCV: 19 matched HCs			
				**p < 0.01**		**p < 0.05**	
			**test count**	**p_uncorr**	**p_FDR**	**p_uncorr**	**p_FDR**
**PVE**							
	raw	count	57	1	0	7	0
		percentage		1.75%	0.00%	12.28%	0.00%
		p_bino (nominal)		0.436	n.a.	0.023	n.a.
		p_bino (empirical)		**0**	1	$<10^{-3}$	1
	eTIV normalized	count	57	1	0	7	0
		percentage		1.75%	0.00%	12.28%	0.00%
		p_bino (nominal)		0.436	n.a.	0.023	n.a.
		p_bino (empirical)		**0**	1	$<10^{-3}$	1
**volume segmentations**							
	raw	count	760	9	0	44	1
		percentage		1.18%	0.00%	5.79%	0.13%
		p_bino (nominal)		0.352	n.a.	0.179	n.a.
		p_bino (empirical)		0.038	0.035	0.053	0.062
	eTIV normalized	count	760	9	0	44	1
		percentage		1.18%	0.00%	5.79%	0.13%
		p_bino (nominal)		0.352	n.a.	0.179	n.a.
		p_bino (empirical)		0.014	0.035	0.046	0.098
	asymmetry	count	266	2	1	12	1
		percentage		0.75%	0.38%	4.51%	0.38%
		p_bino (nominal)		0.503	n.a.	0.427	n.a.
		p_bino (empirical)		0.023	0.429	0.070	0.693
**cortical GM volume**							
	raw	count	4104	70	3	318	4
		percentage		1.71%	0.07%	7.75%	0.10%
		p_bino (nominal)		**$<10^{-4}$**	n.a.	**$<10^{-13}$**	n.a.
		p_bino (empirical)		0.037	0.451	$<10^{-3}$	0.028
	eTIV normalized	count	4104	70	3	321	4

		percentage		1.71%	0.07%	7.82%	0.10%
		p_bino (nominal)		**$<10^{-4}$**	n.a.	**$<10^{-14}$**	n.a.
		p_bino (empirical)		0.051	0.451	$<10^{-3}$	0.028
	asymmetry	count	2052	29	0	156	1
		percentage		1.41%	0.00%	7.60%	0.05%
		p_bino (nominal)		0.044	n.a.	**$<10^{-6}$**	n.a.
		p_bino (empirical)		0.009	1.000	0.019	**0**
**surface area**							
	raw	count	4104	88	2	386	5
		percentage		2.14%	0.05%	9.41%	0.12%
		p_bino (nominal)		**$<10^{-9}$**	n.a.	**0**	n.a.
		p_bino (empirical)		**$<10^{-7}$**	0.349	**$<10^{-13}$**	0.422
	eTIV normalized	count	4104	88	2	391	5
		percentage		2.14%	0.05%	9.53%	0.12%
		p_bino (nominal)		**$<10^{-9}$**	n.a.	**0.000**	n.a.
		p_bino (empirical)		**$<10^{-5}$**	0.349	**$<10^{-11}$**	0.422
	asymmetry	count	2052	37	0	176	1
		percentage		1.80%	0.00%	8.58%	0.05%
		p_bino (nominal)		$<10^{-3}$	n.a.	**$<10^{-11}$**	n.a.
		p_bino (empirical)		0.173	0.327	$<10^{-3}$	0.346
**mean thickness**							
	raw	count	4104	40	0	271	0
		percentage		0.97%	0.00%	6.60%	0.00%
		p_bino (nominal)		0.476	n.a.	**$<10^{-5}$**	n.a.
		p_bino (empirical)		0.002	0.107	**$<10^{-5}$**	***$<10^{-4}$***
	eTIV normalized	count	4104	40	0	271	0
		percentage		0.97%	0.00%	6.60%	0.00%
		p_bino (nominal)		0.476	n.a.	**$<10^{-5}$**	n.a.
		p_bino (empirical)		$<10^{-3}$	0.107	0.256	0.002
	asymmetry	count	2052	24	0	126	0
		percentage		1.17%	0.00%	6.14%	0.00%
		p_bino (nominal)		0.248	n.a.	0.012	n.a.

		p_bino (empirical)		0.259	1.000	0.250	0.327
**SD of thickness**							
	raw	count	4104	28	0	187	1
		percentage		0.68%	0.00%	4.56%	0.02%
		p_bino (nominal)		0.020	n.a.	0.101	n.a.
		p_bino (empirical)		$<10^{-3}$	0.011	0.077	$<10^{-3}$
	eTIV normalized	count	4104	29	0	196	1
		percentage		0.71%	0.00%	4.78%	0.02%
		p_bino (nominal)		0.030	n.a.	0.269	n.a.
		p_bino (empirical)		$<10^{-3}$	0.011	0.061	**$<10^{-3}$**
	asymmetry	count	2052	15	0	136	0
		percentage		0.73%	0.00%	6.63%	0.00%
		p_bino (nominal)		0.130	n.a.	$<10^{-3}$	n.a.
		p_bino (empirical)					
**mean curvature**							
	raw	count	4104	30	0	188	1
		percentage		0.73%	0.00%	4.58%	0.02%
		p_bino (nominal)		0.044	n.a.	0.115	n.a.
		p_bino (empirical)		0.033	0.002	0.057	$<10^{-3}$
	eTIV normalized	count	4104	31	0	197	1
		percentage		0.76%	0.00%	4.80%	0.02%
		p_bino (nominal)		0.063	n.a.	0.293	n.a.
		p_bino (empirical)		0.048	0.002	0.115	$<10^{-3}$
	asymmetry	count	2052	29	0	142	0
		percentage		1.41%	0.00%	6.92%	0.00%
		p_bino (nominal)		0.044	n.a.	$<10^{-4}$	n.a.
		p_bino (empirical)		0.246	0.007	0.200	0.001
**Gaussian curvature**							
	raw	count	4104	87	39	168	46
		percentage		2.12%	0.95%	4.09%	1.12%
		p_bino (nominal)		**$<10^{-9}$**	n.a.	0.003	n.a.

		p_bino (empirical)		0.048	0.007	0.393	$<10^{-3}$
	eTIV normalized	count	4104	89	40	171	48
		percentage		2.17%	0.97%	4.17%	1.17%
		p_bino (nominal)		**$<10^{-10}$**	n.a.	0.007	n.a.
		p_bino (empirical)		0.048	0.005	0.384	$<10^{-3}$
	asymmetry	count	2052	37	8	138	10
		percentage		1.80%	0.39%	6.73%	0.49%
		p_bino (nominal)		$<10^{-3}$	n.a.	$<10^{-3}$	n.a.
		p_bino (empirical)		**$<10^{-4}$**	0.522	0.147	0.024
**folding index**							
	raw	count	4104	129	61	223	76
		percentage		3.14%	1.49%	5.43%	1.85%
		p_bino (nominal)		**0**	n.a.	0.109	n.a.
		p_bino (empirical)		0.284	0.141	0.035	0.248
	eTIV normalized	count	4104	134	63	229	80
		percentage		3.27%	1.54%	5.58%	1.95%
		p_bino (nominal)		**0**	n.a.	0.049	n.a.
		p_bino (empirical)		0.194	0.169	0.037	0.363
	asymmetry	count	2052	43	4	132	7
		percentage		2.10%	0.19%	6.43%	0.34%
		p_bino (nominal)		**$<10^{-5}$**	n.a.	0.002	n.a.
		p_bino (empirical)		0.115	0.057	0.009	0.023
**curvature index**							
	raw	count	4104	85	21	191	30
		percentage		2.07%	0.51%	4.65%	0.73%
		p_bino (nominal)		**$<10^{-8}$**	n.a.	0.163	n.a.
		p_bino (empirical)		0.076	$<10^{-3}$	0.222	$<10^{-3}$
	eTIV normalized	count	4104	87	21	195	30
		percentage		2.12%	0.51%	4.75%	0.73%
		p_bino (nominal)		**$<10^{-9}$**	n.a.	0.245	n.a.
		p_bino (empirical)		0.082	$<10^{-3}$	0.222	$<10^{-3}$
	asymmetry	count	2052	31	3	126	3

	percentage		1.51%	0.15%	6.14%	0.15%
	p_bino (nominal)		0.018	n.a.	0.012	n.a.
	p_bino (empirical)		0.033	0.192	0.220	**$<10^{-3}$**
**grey-white contrast**						
raw	count	4104	0	0	2	0
	percentage		0.00%	0.00%	0.05%	0.00%
	p_bino (nominal)		***$<10^{-17}$***	n.a.	***$<10^{-87}$***	n.a.
	p_bino (empirical)		$<10^{-3}$	1.000	***$<10^{-43}$***	1.000
scanner/sequence corrected	count	4104	0	0	8	0
	percentage		0.00%	0.00%	0.19%	0.00%
	p_bino (nominal)		***$<10^{-17}$***	n.a.	***$<10^{-77}$***	n.a.
	p_bino (empirical)		$<10^{-3}$	1.000	***$<10^{-35}$***	1.000
asymmetry	count	2052	32	1	130	2
	percentage		1.56%	0.05%	6.34%	0.10%
	p_bino (nominal)		0.011	n.a.	0.004	n.a.
	p_bino (empirical)		0.104	0.346	0.086	0.471

Table S4B:
Parameter-specific anomalies detected in the patient-matched LOOCV test set at two significance levels α = 0.01 and α = 0.05. In addition to a test against the expected error rate α (binomial tests) the 19 datasets were also tested against the empirical anomaly rates obtained in the randomly selected subset (Table S4a). The table arrangement corresponds exactly to that of Table 3 of the main text.
Abbreviations: FDR, false discovery rate; LOOCV, leave-one-out cross-validation; n.a., not applicable.

Table S4C

			2 MS patients / 19 MRIs			
			p < 0.01		p < 0.05	
		test count	p_uncorr	p_FDR	p_uncorr	p_FDR
**PVE**						
raw	count	57	0	0	0	0
	percentage		0.00%	0.00%	0.00%	0.00%
	p_bino (nominal)		0.563	n.a.	0.054	n.a.
	p_bino (empirical)		1	1	0.323	1
eTIV normalized	count	57	0	0	0	0
	percentage		0.00%	0.00%	0.00%	0.00%
	p_bino (nominal)		0.563	n.a.	0.054	n.a.
	p_bino (empirical)		1	1	0.323	1
**volume segmentations**						
raw	count	760	75	14	201	31
	percentage		9.87%	1.84%	26.45%	4.08%
	p_bino (nominal)		**0**	n.a.	**0**	n.a.
	p_bino (empirical)		**0**	**$<10^{-4}$**	**0**	**$<10^{-15}$**
eTIV normalized	count	760	78	15	209	32
	percentage		10.26%	1.97%	27.50%	4.21%
	p_bino (nominal)		**0**	n.a.	**0**	n.a.
	p_bino (empirical)		**0**	**$<10^{-5}$**	**0**	**0**
asymmetry	count	266	29	12	50	18
	percentage		10.90%	4.51%	18.80%	6.77%
	p_bino (nominal)		**0**	n.a.	**$<10^{-15}$**	n.a.
	p_bino (empirical)		**$<10^{-9}$**	**$<10^{-12}$**	**$<10^{-9}$**	**$<10^{-15}$**
**cortical GM volume**						
raw	count	4104	94	2	403	12
	percentage		2.29%	0.05%	9.82%	0.29%
	p_bino (nominal)		**$<10^{-12}$**	n.a.	**0**	n.a.
	p_bino (empirical)		**$<10^{-5}$**	0.251	**0**	0.310
eTIV normalized	count	4104	94	2	408	12

		percentage		2.29%	0.05%	9.94%	0.29%
		p_bino (nominal)		**$<10^{-12}$**	n.a.	**0**	n.a.
		p_bino (empirical)		**$<10^{-5}$**	0.251	**0**	0.310
	asymmetry	count	2052	39	6	150	14
		percentage		1.90%	0.29%	7.31%	0.68%
		p_bino (nominal)		$<10^{-3}$	n.a.	**$<10^{-5}$**	n.a.
		p_bino (empirical)		**$<10^{-5}$**	**0**	0.059	**0**
**surface area**							
	raw	count	4104	79	8	341	18
		percentage		1.92%	0.19%	8.31%	0.44%
		p_bino (nominal)		**$<10^{-7}$**	n.a.	**0**	n.a.
		p_bino (empirical)		**$<10^{-4}$**	0.021	**$<10^{-6}$**	**$<10^{-4}$**
	eTIV normalized	count	4104	80	8	345	18
		percentage		1.95%	0.19%	8.41%	0.44%
		p_bino (nominal)		**$<10^{-7}$**	n.a.	**0**	n.a.
		p_bino (empirical)		$<10^{-4}$	0.021	**$<10^{-4}$**	**$<10^{-4}$**
	asymmetry	count	2052	56	6	212	10
		percentage		2.73%	0.29%	10.33%	0.49%
		p_bino (nominal)		**$<10^{-10}$**	n.a.	**0**	n.a.
		p_bino (empirical)		**$<10^{-4}$**	0.001	**$<10^{-9}$**	**$<10^{-3}$**
**mean thickness**							
	raw	count	4104	162	2	585	25
		percentage		3.95%	0.05%	14.25%	0.61%
		p_bino (nominal)		**0**	n.a.	**0**	n.a.
		p_bino (empirical)		**0**	0.613	**0**	**$<10^{-3}$**
	eTIV normalized	count	4104	167	2	605	25
		percentage		4.07%	0.05%	14.74%	0.61%
		p_bino (nominal)		**0**	n.a.	**0**	n.a.
		p_bino (empirical)		**0**	0.613	**0**	**$<10^{-8}$**
	asymmetry	count	2052	53	5	202	11
		percentage		2.58%	0.24%	9.84%	0.54%
		p_bino (nominal)		**$<10^{-8}$**	n.a.	**0**	n.a.

		p_bino (empirical)		**<10⁻8**	**0**	**<10⁻12**	**<10⁻7**
**SD of thickness**							
	raw	count	4104	42	2	254	6
		percentage		1.02%	0.05%	6.19%	0.15%
		p_bino (nominal)		0.461	n.a.	<10⁻3	n.a.
		p_bino (empirical)		0.103	0.177	<10⁻3	0.126
	eTIV normalized	count	4104	46	2	272	6
		percentage		1.12%	0.05%	6.63%	0.15%
		p_bino (nominal)		0.238	n.a.	**<10⁻5**	n.a.
		p_bino (empirical)		0.145	0.177	**<10⁻3**	0.096
	asymmetry	count	2052	60	1	212	6
		percentage		2.92%	0.05%	10.33%	0.29%
		p_bino (nominal)		**<10⁻12**	n.a.	**0**	n.a.
		p_bino (empirical)					
**mean curvature**							
	raw	count	4104	172	33	420	71
		percentage		4.19%	0.80%	10.23%	1.73%
		p_bino (nominal)		**0**	n.a.	**0**	n.a.
		p_bino (empirical)		**0**	**<10⁻13**	**0**	**0**
	eTIV normalized	count	4104	178	33	437	72
		percentage		4.34%	0.80%	10.65%	1.75%
		p_bino (nominal)		**0**	n.a.	**0**	n.a.
		p_bino (empirical)		**0**	**<10⁻13**	**0**	**0**
	asymmetry	count	2052	100	14	241	27
		percentage		4.87%	0.68%	11.74%	1.32%
		p_bino (nominal)		**0**	n.a.	**0**	n.a.
		p_bino (empirical)		**0**	**0**	**<10⁻11**	**<10⁻8**
**Gaussian curvature**							
	raw	count	4104	331	177	506	223
		percentage		8.07%	4.31%	12.33%	5.43%
		p_bino (nominal)		**0**	n.a.	**0**	n.a.

		p_bino (empirical)		**0**	**0**	**0**	**0**
	eTIV normalized	count	4104	342	183	528	231
		percentage		8.33%	4.46%	12.87%	5.63%
		p_bino (nominal)		**0**	n.a.	**0**	n.a.
		p_bino (empirical)		**0**	**0**	**0**	**0**
	asymmetry	count	2052	174	56	310	78
		percentage		8.48%	2.73%	15.11%	3.80%
		p_bino (nominal)		**0**	n.a.	**0**	n.a.
		p_bino (empirical)		**0**	**0**	**0**	**0**
**folding index**							
	raw	count	4104	292	195	427	229
		percentage		7.12%	4.75%	10.40%	5.58%
		p_bino (nominal)		**0**	n.a.	**0**	n.a.
		p_bino (empirical)		**0**	**0**	**0**	**0**
	eTIV normalized	count	4104	303	203	442	238
		percentage		7.38%	4.95%	10.77%	5.80%
		p_bino (nominal)		**0**	n.a.	**0**	n.a.
		p_bino (empirical)		**0**	**0**	**0**	**0**
	asymmetry	count	2052	163	52	300	86
		percentage		7.94%	2.53%	14.62%	4.19%
		p_bino (nominal)		**0**	n.a.	**0**	n.a.
		p_bino (empirical)		**0**	**0**	**0**	**0**
**curvature index**							
	raw	count	4104	210	95	369	120
		percentage		5.12%	2.31%	8.99%	2.92%
		p_bino (nominal)		**0**	n.a.	**0**	n.a.
		p_bino (empirical)		**0**	**$<10^{-12}$**	**0**	**$<10^{-15}$**
	eTIV normalized	count	4104	215	96	379	122
		percentage		5.24%	2.34%	9.23%	2.97%
		p_bino (nominal)		**0**	n.a.	**0**	n.a.
		p_bino (empirical)		**0**	**$<10^{-13}$**	**0**	**0**
	asymmetry	count	2052	119	18	292	47

		percentage		5.80%	0.88%	14.23%	2.29%
		p_bino (nominal)		**0**	n.a.	**0**	n.a.
		p_bino (empirical)		**0**	**$<10^{-5}$**	**0**	**$<10^{-10}$**
**grey-white contrast**						
raw	count	4104	3	0	27	1
	percentage		0.07%	0.00%	0.66%	0.02%
	p_bino (nominal)		***$<10^{-13}$***	n.a.	***$<10^{-56}$***	n.a.
	p_bino (empirical)		0.048	1.000	***$<10^{-20}$***	**0**
scanner/sequence corrected	count	4104	27	1	111	8
	percentage		0.66%	0.02%	2.70%	0.19%
	p_bino (nominal)		0.013	n.a.	***$<10^{-13}$***	n.a.
	p_bino (empirical)		**$<10^{-7}$**	**0**	0.414	**0**
asymmetry	count	2052	86	13	287	32
	percentage		4.19%	0.63%	13.99%	1.56%
	p_bino (nominal)		**0**	n.a.	**0**	n.a.
	p_bino (empirical)		**0**	**$<10^{-6}$**	**0**	**0**

Table S4C:
Parameter-specific anomalies detected in the datasets of the MS patients 2 and 3 at two significance levels α = 0.01 and α = 0.05. In addition to a test against the expected error rate α (binomial tests) the 19 datasets were also tested against the empirical anomaly rates obtained in the randomly selected subset (Table S4a). The table arrangement corresponds exactly to that of Table 3 of the main text.
Abbreviations: FDR, false discovery rate; LOOCV, leave-one-out cross-validation; n.a., not applicable.

Supplementary references

Dale AM, Fischl B and Sereno MI. 1999 Cortical surface–based analysis. I. Segmentation and surface reconstruction. NeuroImage **9** 179

Deichmann R, Schwarzbauer C, Turner, R. 2004 Optimisation of the 3D MDEFT sequence for anatomical brain imaging: technical implications at 1.5 and 3 T. NeuroImage **21** 757–67

Desikan R S et al. 2006 An automated labeling system for subdividing the human cerebral cortex on MRI scans into gyral based regions of interest. NeuroImage 31 968–980

Destrieux C, Fischl B, Dale A and Halgren E 2010 Automatic parcellation of human cortical gyri and sulci using standard anatomical nomenclature. NeuroImage 53 1–15

Fischl B, Sereno MI and Dale AM. 1999 Cortical surface-based analysis. II: Inflation, flattening, and a surface-based coordinate system. NeuroImage **9** 195–207

Fischl B, Sereno MI, Tootell RB and Dale AM. 1999 High-resolution intersubject averaging and a coordinate system for the cortical surface. Hum. Brain Mapp. **8** 272–84

Fischl B and Dale AM. 2000 Measuring the thickness of the human cerebral cortex from magnetic resonance images. Proc. Natl. Acad. Sci. U.S.A. **97** 11050–5

Fischl B, et al. 2002 Whole brain segmentation: automated labeling of neuroanatomical structures in the human brain. Neuron **33** 341–55

Fischl B, et al. 2004 Sequence–independent segmentation of magnetic resonance images. NeuroImage **23** (Suppl 1) S69–S84

Held P, Fellner C, Fellner F, Geissler A. and Gmeinwieser J. 1995 Three-dimensional MP-RAGE – an alternative to conventional three-dimensional FLASH sequences for the diagnosis of viscerocranial tumours? Br. J. Radiol. **68** 1316–24.

Jack CR Jr, et al. 2008 The Alzheimer's Disease Neuroimaging Initiative (ADNI): MRI methods. J. Magn. Reson. Imaging **27** 685–91

van der Kouwe AJ, Benner T, Salat DH and Fischl, B. 2008 Brain morphometry with multiecho MPRAGE. NeuroImage **40** 559–69

Pienaar R, Fischl B, Caviness V, Makris N and Grant PE. 2008 A methodology for analyzing curvature in the developing brain from preterm to adult. Int. J. Imaging Syst. Technol. **18** 42–68

Salat DH, et al. 2009 Age-associated alterations in cortical gray and white matter signal intensity and gray to white matter contrast. NeuroImage **48** 21–28

Smith SM. 2002 Fast robust automated brain extraction. Human Brain Mapp. **17** 143–55

Smith SM, et al. 2004 Advances in functional and structural MR image analysis and implementation as FSL. NeuroImage **23** (Suppl. 1) S208–S219

Winkler AM, Kochunov P, Blangero J, Almasy L, Zilles K, Fox PT, Duggirala R and Glahn DC 2010 Cortical thickness or grey matter volume? The importance of selecting the phenotype for imaging genetics studies. NeuroImage 53 1135–46

Zhang Y, Brady M and Smith S. 2001 Segmentation of brain MR images through a hidden Markov random field model and the expectation–maximization algorithm. IEEE Trans. Med. Imag. **20** 45–57
